# HELQ is a dual-function DSB repair enzyme modulated by RPA and RAD51

**DOI:** 10.1038/s41586-021-04261-0

**Published:** 2021-12-22

**Authors:** Roopesh Anand, Erika Buechelmaier, Ondrej Belan, Matthew Newton, Aleksandra Vancevska, Artur Kaczmarczyk, Tohru Takaki, David S. Rueda, Simon N. Powell, Simon J. Boulton

**Affiliations:** 1 DSB Repair Metabolism Laboratory, The Francis Crick Institute, London, UK; 2 Memorial Sloan Kettering Cancer Center, New York, NY USA; 3 Graduate School of Medical Sciences, Weill Cornell Medicine, New York, NY USA; 4 Department of Infectious Disease, Faculty of Medicine, Imperial College London, London, UK; 5 Single Molecule Imaging Group, MRC-London Institute of Medical Sciences, London, UK

**Keywords:** Enzyme mechanisms, Single-molecule biophysics, Double-strand DNA breaks

## Abstract

DNA double-stranded breaks (DSBs) are deleterious lesions, and their incorrect repair can drive cancer development^1^. HELQ is a superfamily 2 helicase with 3′ to 5′ polarity, and its disruption in mice confers germ cells loss, infertility and increased predisposition to ovarian and pituitary tumours^2–4^. At the cellular level, defects in HELQ result in hypersensitivity to cisplatin and mitomycin C, and persistence of RAD51 foci after DNA damage^3,5^. Notably, HELQ binds to RPA and the RAD51-paralogue BCDX2 complex, but the relevance of these interactions and how HELQ functions in DSB repair remains unclear^3,5,6^. Here we show that HELQ helicase activity and a previously unappreciated DNA strand annealing function are differentially regulated by RPA and RAD51. Using biochemistry analyses and single-molecule imaging, we establish that RAD51 forms a complex with and strongly stimulates HELQ as it translocates during DNA unwinding. By contrast, RPA inhibits DNA unwinding by HELQ but strongly stimulates DNA strand annealing. Mechanistically, we show that HELQ possesses an intrinsic ability to capture RPA-bound DNA strands and then displace RPA to facilitate annealing of complementary sequences. Finally, we show that HELQ deficiency in cells compromises single-strand annealing and microhomology-mediated end-joining pathways and leads to bias towards long-tract gene conversion tracts during homologous recombination. Thus, our results implicate HELQ in multiple arms of DSB repair through co-factor-dependent modulation of intrinsic translocase and DNA strand annealing activities.

## Abstract (web summary)

HELQ is differentially regulated by RAD51, which stimulates helicase activity, and RPA, which inhibits helicase activity and stimulates annealing.

## Main

**Fig. 1:**
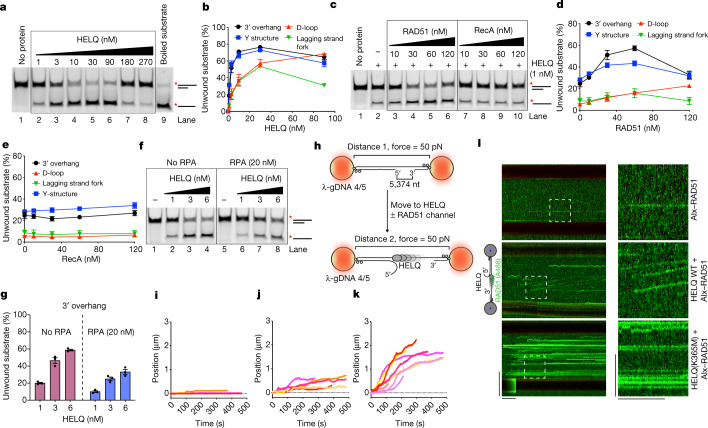
RAD51 forms a co-complex with HELQ and stimulates HELQ unwinding activity. **a**, Representative gel of the DNA unwinding assay with the indicated concentrations of HELQ with 3′ overhang substrate. The asterisk indicates the position of fluorescein isothiocyanate (FITC) labelling at 5′ end of the oligo. The products were resolved on a 10% native polyacrylamide gel. **b**, Quantification of the experiments such as shown in **a** and Extended Data Fig. 1b–d. HELQ concentrations of only 1–90 nM are shown. *n* = 4 independent experiments. Data are mean ± s.e.m. **c**, Representative gel of DNA unwinding of 3′ overhang substrate with HELQ and the indicated concentrations of RAD51 or RecA. **d**, **e**, Quantification of the experiments shown in **c** and Extended Data Fig. 2c, d for RAD51 (**d**) and RecA (**e**). *n* = 3 (3′ overhang), *n* = 4 (Y-structure), *n* = 3 (D-loop) and *n* = 3 (lagging strand fork) independent experiments. Data are mean ± s.e.m. **f**, Representative gel of the DNA unwinding assay of 3′ overhang substrate with the indicated concentrations of HELQ in the absence and presence of RPA (20 nM). **g**, Quantification of the experiments shown in **f**. *n* = 3 independent experiments. Data are mean ± s.e.m. **h**, Schematics of the experimental set-up of the optical tweezer (C-Trap) system to observe DNA unwinding. These experiments were performed at room temperature. **i**–**k**, Bead centre displacement measured between the traps as a function of time with 25 nM RAD51 (**i**), 50 nM HELQ (**j**), and 25 nM RAD51 and 50 nM HELQ (**k**). The traces represent individual DNA molecules (*n* ≥ 4). **l**, Representative kymographs of single Alx–RAD51 binding events on gapped DNA in the presence or absence of 50 nM HELQ or HELQ(K365M). Unidirectional movement of Alx–RAD51 indicates translocation of Alx–RAD51–HELQ complex. Scale bars, 60 s (horizontal), 10 µm (vertical, left), 5 µm (vertical, right).

**Extended Data Fig. 1:**
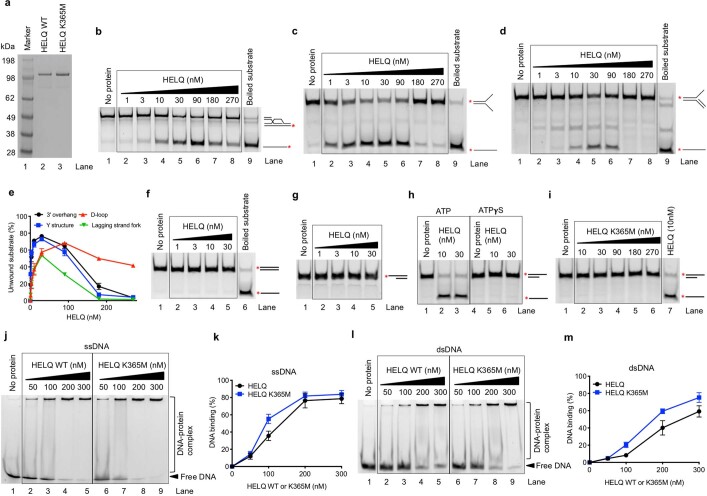
HELQ specifically unwinds substrates with 3′ overhang and D-loop. **a**, SDS-PAGE gel (4–12% polyacrylamide) showing purified recombinant human HELQ WT and HELQ(K365M) from insect cells. The gel was stained with Coomassie brilliant blue (CBB). We used two and single preparations of HELQ WT and HELQ(K365M) respectively in this study. **b**-**d**, Representative native gels (10% polyacrylamide) of DNA unwinding assay of D-loop, Y-structure and lagging strand fork with indicated concentrations of HELQ. The asterisk indicates the position of FITC labelling at 5′ end of oligo. **e**, Quantification of experiments such as shown in b-d and Fig. 1a with HELQ concentration ranging from 1-270 nM. n= 4 independent experiments; mean ± S.E.M. **f-g**, Representative native gels of DNA unwinding assay of dsDNA and 5′-overhang with indicated concentrations of HELQ. **h**, Native gel showing the DNA unwinding assay of 3′-overhang by HELQ in the presence of ATP (2 mM) and ATPγS (2 mM), a poorly hydrolysable ATP analogue (n=2). **i**, Native gel showing DNA unwinding assay of 3′-overhang with indicated concentrations of HELQ(K365M) (n =2). **j**, Electrophoretic mobility shift assay (EMSA) with ssDNA and indicated concentrations of HELQ and HELQ(K365M). The final products were resolved with native 6% polyacrylamide gels. **k**, Quantification of experiments such as shown in j. n= 3 independent experiments; mean ± S.E.M. **l**, EMSA with dsDNA and indicated concentrations of HELQ and HELQ(K365M). **m**, Quantification of experiments such as shown in l. n= 3 independent experiments; mean ± S.E.M.

To investigate the functions of HELQ, we purified recombinant human HELQ from insect cells (Extended Data Fig. 1a), which efficiently unwound substrates containing 3′ overhangs or a D-loop (Fig. 1a, b and Extended Data Fig. 1b–d). As previously reported, HELQ prefers to unwind single and double-stranded DNA junctions and therefore showed greater unwinding of 3′ overhangs and Y-structures than 3′ lagging strand forks and D-loops^7^. However, at higher concentrations of HELQ, no unwound product was observed, especially for substrates containing 3′ overhangs (described below; Extended Data Fig. 1e). HELQ showed no unwinding with ATPγS, a poorly hydrolysable ATP analogue, and failed to unwind dsDNA and 5′ overhang substrates (Extended Data Fig. 1f–h). The helicase-dead mutant of HELQ (HELQ(K365M)) lacked DNA-unwinding activity and showed slightly increased binding to ssDNA and dsDNA compared with the wild-type (WT) protein (Extended Data Fig. 1a, i–m).

## RAD51 stimulates HELQ unwinding activity

**Extended Data Fig. 2:**
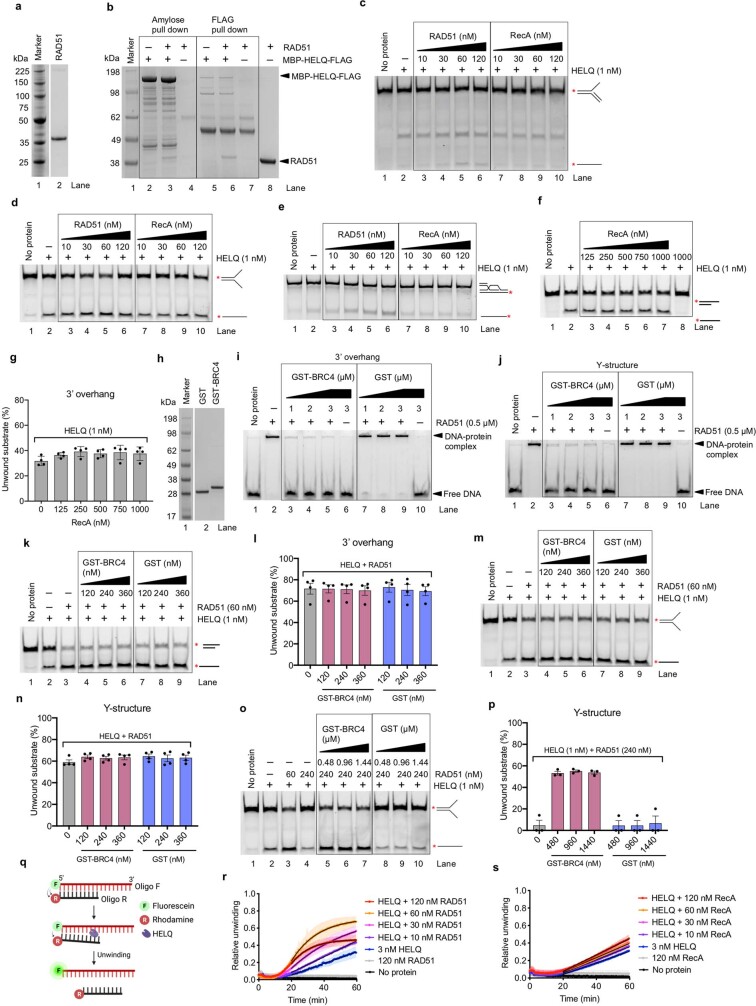
RAD51 interacts directly to HELQ and promotes its helicase activity. **a**, SDS-PAGE gel (4-12%) showing purified recombinant human RAD51 from *E. coli*. The gel was stained with CBB. We used two preparations of RAD51 in this study. **b**, Protein interaction analysis of MBP-HELQ-Flag and RAD51 using both amylose and Flag pull-down assay. The final eluates were run on SDS-PAGE gel (4-12%) and stained with CBB. The interaction analysis was repeated 3 times with similar results. **c-e**, Representative native gels of DNA unwinding of D-loop, Y-structure and lagging strand fork substrates with HELQ (1 nM) and indicated concentrations of either RAD51 or RecA. **f**, Representative native gel of DNA unwinding of 3′-overhang with indicated concentrations of RecA and HELQ (1 nM). **g**, Quantification of experiments such as shown in f. n = 4 independent experiments; mean ± S.E.M. **h**, SDS-PAGE gel (4-12%) showing purified recombinant GST and GST-BRC4 peptides from *E. coli*. The gel was stained with CBB. Single preparation of GST and GST-BRC4 used in this study. **i-j**, EMSA gels (6% polyacrylamide) showing RAD51 binding to 3′-overhang and Y-structure in the presence and absence of indicated concentration of GST-BRC4 and GST peptides. **k**, Representative native gel of DNA unwinding of 3′-overhang by HELQ with RAD51 and indicated concentrations of GST-BRC4 and GST peptides. **l**, Quantification of experiments such as shown in k. n = 4 independent experiments; mean ± S.E.M. **m**, Representative native gel of DNA unwinding of Y-structure by HELQ with RAD51 and indicated concentrations of GST-BRC4 and GST peptides. **n**, Quantification of experiments such as shown in m. n = 4 independent experiments; mean ± S.E.M. **o**, Representative native gel of DNA unwinding of Y-structure by HELQ with inhibitory concentration of RAD51 and indicated concentrations of for substrates GST-BRC4 and GST peptides. **p**, Quantification of experiments such as shown in o. n = 3 independent experiments; mean ± S.E.M. **q**, Schematics representation of quenching-based kinetic DNA unwinding assay of 3′-overhang. Initially, oligo F (49-mer), labelled at 5′ end with fluorescein (F), is annealed with oligo R, which is labelled with rhodamine (R) at 3′ end. Due to close proximity, FRET from fluorescein is quenched by rhodamine constitutively resulting in low FRET signal. Upon DNA unwinding, DNA strands are separated and thus rhodamine no longer able to quench fluorescein, results in higher FRET signal. **r**, Relative unwinding of 3′-overhang with HELQ and indicated concentrations of RAD51 as determined by quenching-based kinetic assay. n = 3 independent experiments; shaded area represents mean ± S.E.M.; black lines represent exponential or linear fits. **s**, Relative unwinding of 3′-overhang with HELQ and indicated concentrations of RecA as determined by quenching-based kinetic assay. n = 3 independent experiments; shaded area represents mean ± S.E.M.; black lines represent exponential or linear fits.

**Extended Data Fig. 3:**
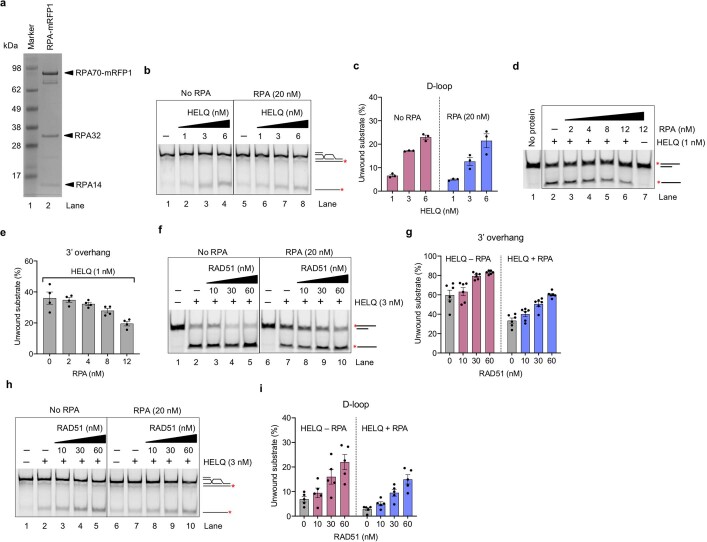
RPA inhibits HELQ DNA unwinding activity. **a**, SDS-PAGE gel (4-12%) showing purified recombinant human RPA-mRFP1 from *E. coli*. The gel was stained with CBB. Single preparation of RPA-mRFP1 was used in this study. **b**, Representative gel of DNA unwinding assay of D-loop with HELQ in the absence and presence of RPA (20 nM). **c**, Quantification of experiments such as shown in b. n = 3 independent experiments; mean ± S.E.M. **d**, Representative gel of DNA unwinding assay of 3′-overhang with HELQ (1 nM) and indicated concentrations of RPA. **e**, Quantification of experiments such as shown in d. n = 3 independent experiments; mean ± S.E.M. **f**, Representative gel of DNA unwinding assay of 3′-overhang with HELQ (3 nM) and indicated concentrations of RAD51, in the absence and presence of RPA (20 nM). **g**, Quantification of experiments such as shown in f. n = 6 independent experiments; mean ± S.E.M. **h**, Representative gel of DNA unwinding assay of D-loop with HELQ (3 nM) and indicated concentrations of RAD51, in absence and presence of RPA (20 nM). **i**, Quantification of experiments such as shown in h. n = 5 independent experiments; mean ± S.E.M.

In vivo studies have shown that HELQ-deficient cells exhibit persistent RAD51 foci after DNA damage^3,8^. Furthermore, HELQ-1 from *Caenorhabditis elegans* interacts with RAD-51 (ref. ^8^). HELQ and human RAD51, purified from *Escherichia coli* (Extended Data Fig. 2a), also interact directly (Extended Data Fig. 2b). In unwinding assays, RAD51 strongly stimulated HELQ helicase activity with all of the tested substrates, whereas bacterial RecA—an orthologue of RAD51—did not stimulate HELQ even at higher concentrations (Fig. 1c–e and Extended Data Fig. 2c–g). We next purified and tested the BRC4 peptide (single BRCA2 BRC repeat), which prevents RAD51 from binding to DNA (Extended Data Fig. 2h–j). The BRC4 peptide did not inhibit stimulation of HELQ by RAD51(Extended Data Fig. 2k–n), indicating that RAD51 DNA binding is not required for HELQ stimulation and excludes DNA sequestration as a possible mechanism for stimulation by RAD51. We observed that, at a higher concentration of RAD51 (that is, 120 nM), HELQ unwinding activity is inhibited (Fig. 1c, d). To investigate this, we tested the BRC4 peptide with an excess RAD51 and found that inhibiting RAD51 DNA binding also rescued DNA unwinding by HELQ (Extended Data Fig. 2o, p). Furthermore, by measuring the kinetics of DNA unwinding by HELQ, we found that addition of RAD51 resulted in a concentration-dependent increase in the HELQ DNA unwinding rate, whereas addition of RecA had no effect (Extended Data Fig. 2q–s). In cells, ssDNA generated during DNA processing is bound by RPA. To mimic these conditions, we purified fluorescently tagged human RPA–mRFP1 from *E. coli* (Extended Data Fig. 3a). Addition of RPA inhibited DNA unwinding by HELQ, especially for 3′ overhang substrates (Fig. 1f, g and Extended Data Fig. 3b, c). At lower concentrations that were insufficient to cover the entire ssDNA region, RPA still inhibited HELQ unwinding of 3′ overhang substrates (Extended Data Fig. 3d, e). Despite the inhibitory effect of RPA, RAD51 still stimulated HELQ helicase activity in the presence of RPA (Extended Data Fig. 3f–i).

## Visualization of HELQ DNA unwinding

**Extended Data Fig. 4:**
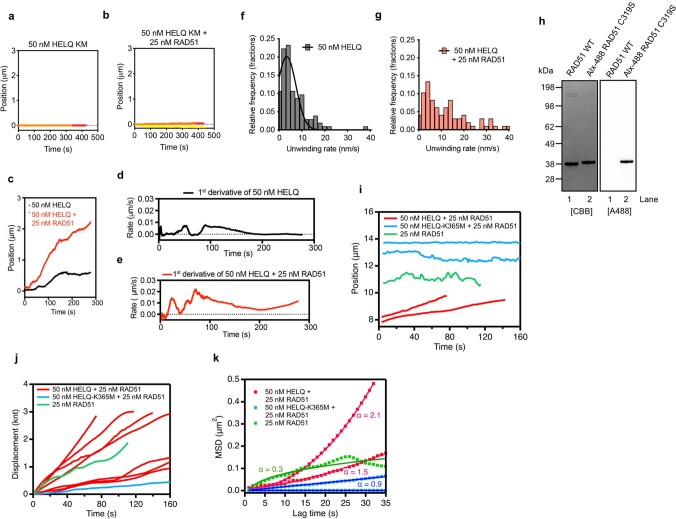
RAD51–HELQ complex translocates along ssDNA backbone. **a**–**b**. Bead centre displacement measured between the traps as a function of time in indicated conditions. Traces represent individual DNA molecules (n ≥ 3). **c**. Example of two real-time bead displacement traces for indicated conditions. Unwinding bursts of linearly increasing bead distance are interspersed by pauses where no distance change is observed. **d**-**e**. First derivative with applied smoothening of traces shown in **c**. Height of individual peaks corresponds to the rate of individual unwinding bursts. **f**–**g**. Histogram showing HELQ unwinding burst rate distribution in the absence (n = 103) and presence (n = 97) of RAD51. Black line in **f** represents single Gaussian fits. **h**. Chemical labelling of RAD51. RAD51 C319S was labelled in pH 7.0 using maleimide esters of Alexa Fluor 488. After reaction termination and purification of labelled species, labelling efficiency was assessed, and free dye component was evaluated using SDS-PAGE (4-12%) and subsequent fluorescent imaging. 1:1 labelling stoichiometry was achieved as measured. spectrophotometrically. Proteins were labelled typically with 80-100% labelling efficiency. Single preparation of RAD51 C319S was used in this study. **i**, Displacement of proteins bound to individual DNA tethers. The representative trajectories illustrate either unidirectional movement (red), 1D diffusion (green) and static binding with occasional diffusion (blue). The positions of molecules in time were measured by fitting a moving window of three kymograph frames with Gaussian function. **j**, Total displacement of translocating HELQ-Alx–RAD51 complexes (red) used to calculate the translocation rate. Two populations of translocating molecules can be distinguished: faster with the mean rate of 14 ± 5 nm/s and slower with the mean rate of 4 ± 1 nm/s. The slower rate might result due to “pushing” of sparsely bound Alx–RAD51 on DNA by HELQ alone species. The total displacement of a representative RAD51 filament (green) includes all frame-to-frame displacements, regardless of directionality. The stationary molecule apparent displacement (blue) of 2 ± 1 nm/s results from thermal fluctuations of the tethered DNA. **k**, Mean Square Displacement calculated from the trajectories shown in panel i, plotted as a function of time interval for a period up to 35 sec. Fitting the MSD plot with a power law *D∆t*^*α*^ resulted in the factor α > 1 for HELQ-RAD51 complexes (red) indicating their diffusive (directed) motion, in contrast to diffusing RAD51 and HELQ(K365M)-RAD51 complexes (α ≤ 1) (green, blue).

To better understand HELQ stimulation by RAD51, we used an optical tweezer set-up combined with microfluidics and confocal microscopy (C-TRAP) for single-molecule imaging (SMI) analysis. As shown in Fig 1h, a single dsDNA molecule (λ-DNA) containing a ssDNA gap^9^ was tethered between two optically trapped beads and held at constant force (50 pN) to prevent the reannealing of unwound DNA. After addition of HELQ, DNA unwinding was observed as an increase in the distance between the beads due to the expansion of the ssDNA region. Neither RAD51 alone nor HELQ(K365M) showed evidence of unwinding (Fig. 1i and Extended Data Fig. 4a). Combining WT HELQ and RAD51 resulted in a considerable increase in overall DNA unwinding, whereas no such stimulation was observed with HELQ(K365M) (Fig. 1j, k and Extended Data Fig. 4b). Within unwinding traces for individual DNA molecules, rapid unwinding bursts interspersed by pauses can be distinguished (Extended Data Fig. 4c–e) and corresponded to a mean rate of 3.3 ± 0.4 nm s^−1^ (mean ± s.e.m Extended Data Fig. 4f). In the presence of RAD51, an increased number of molecules showed greater unwinding rates (Extended Data Fig. 4g). To directly visualize RAD51 during DNA unwinding with HELQ, mutant RAD51(C319S) was purified and efficiently labelled with Alexa Fluor 488 C5 maleimide dye (Alx–RAD51) (Extended Data Fig. 4h). Whereas Alx–RAD51 alone displayed mostly static binding traces with occasional diffusing species, addition of HELQ showed unidirectionally translocating traces indicating active movement of an Alx–RAD51–HELQ complex along the ssDNA backbone (Fig. 1l and Extended Data Fig. 4i). After analysis, we found that HELQ with RAD51 translocates fastest at the rate of 14 ± 5 nm s^−1^ in gapped substrate (Extended Data Fig. 4j, k). By contrast, HELQ(K365M) retained the ability to bind to RAD51 but showed no translocation with only static or diffusing traces. Together, these results indicate that RAD51 and HELQ form a complex that unwinds DNA at a rate of approximately threefold faster compared with HELQ alone.

## DNA strand annealing by HELQ

**Fig. 2:**
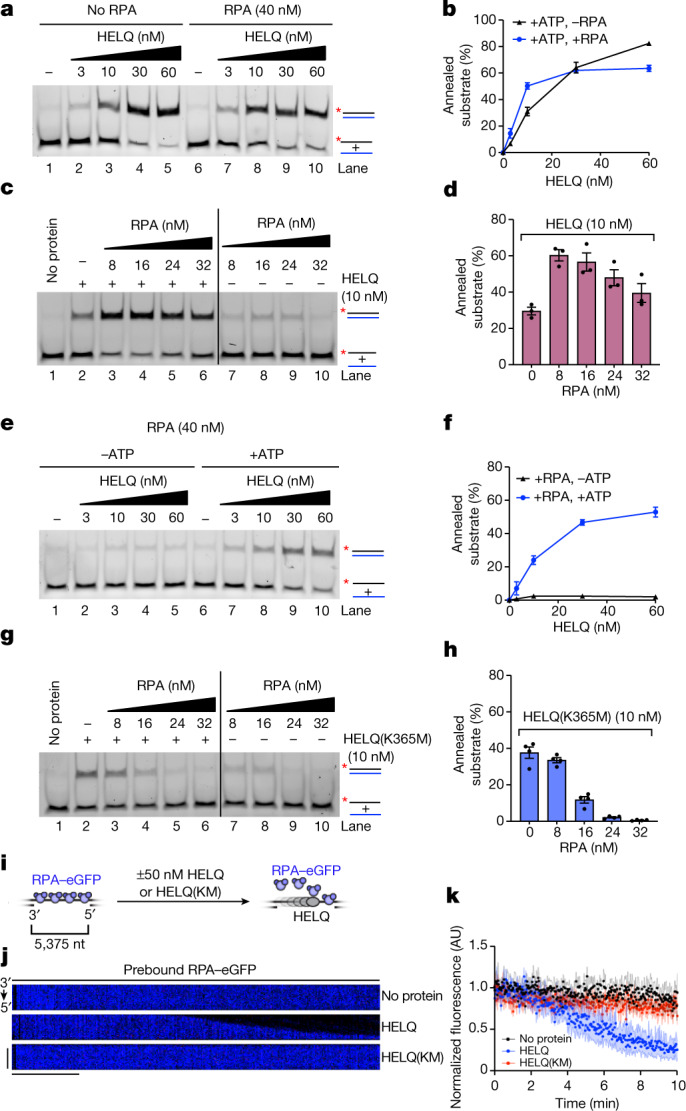
HELQ strips RPA from and anneals ssDNA. **a**, Representative gel of DNA annealing assay with the indicated concentrations of HELQ in the absence and presence of RPA (40 nM). The black and blue colours of substrate represent complementary DNA strands. The asterisk indicates the position of FITC labelling at the 5′ end of the oligo. The products were resolved with 10% native polyacrylamide gel. **b**, Quantification of experiments such as shown in **a**. *n* = 6 independent experiments. Data are mean ± s.e.m. **c**, Representative gel of the DNA annealing assay with HELQ (10 nM) and the indicated concentrations of RPA. **d**, Quantification of the experiments shown in **c**. *n* = 3 independent experiments. Data are mean ± s.e.m. **e**, Representative gel of the DNA annealing assay with the indicated concentrations of HELQ and RPA (40 nM), in the absence and presence of ATP. **f**, Quantification of the experiments shown in **e**. *n *= 3 independent experiments. Data are mean ± s.e.m. **g**, Representative gel of DNA annealing assay with HELQ(K365M) (10 nM) and the indicated concentrations of RPA. **h**, Quantification of the experiments shown in **g**. *n* = 4 independent experiments. Data are mean ± s.e.m. **i**, Schematics of the experimental set-up of the optical tweezer (C-Trap) system to observe RPA–eGFP stripping from ssDNA. These experiments were performed at room temperature. **j**, Representative kymographs of stripping of RPA–eGFP prebound to gapped DNA in the presence or absence of 50 nM HELQ or HELQ(K365M). Unidirectional stripping of RPA–eGFP with HELQ WT indicates 3′ to 5′ translocation of HELQ. **k**, Removal of RPA–eGFP measured from gapped ssDNA as function of time in indicated conditions. The traces represent individual DNA molecules (*n* ≥ 3). Scale bars, 2 min (horizontal), 2 µm (vertical).

**Extended Data Fig. 5:**
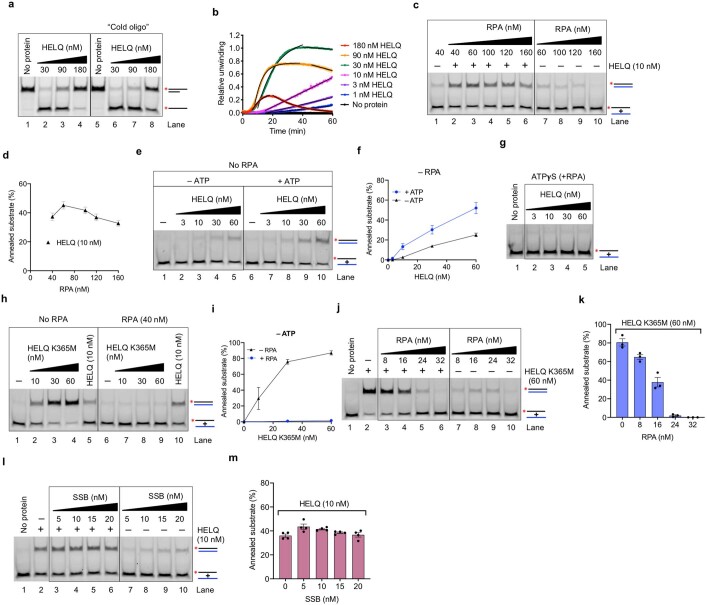
ATP is important for HELQ annealing activity in presence of RPA. **a**, Representative gel of DNA unwinding assay of 3′-overhang with HELQ, in the presence and absence of “cold oligo” (25 nM) i.e., unlabelled oligo with identical DNA sequence as FITC-labelled oligo. **b**, Relative unwinding of 3′ overhang with indicated concentrations of HELQ as determined by quenching-based kinetic assay. n = 3 independent experiments; shaded area represents mean ± S.E.M.; black lines represent exponential or linear fits. **c**, Representative gel of DNA annealing assay with HELQ (10 nM) and indicated concentrations of RPA. The black and blue colours of substrate represent complementary DNA strands. The asterisk indicates the position of FITC labelling at 5′ end. The products were resolved on 10% native polyacrylamide gel. **d**, Quantification of experiments such as shown in c. n = 5 independent experiments; mean ± S.E.M. **e**, Representative gel of DNA annealing assay with indicated concentrations of HELQ in the absence and presence of ATP. **f**, Quantification of experiments such as shown in e. n= 3 independent experiments; mean ± S.E.M. **g**, Native gel showing DNA annealing assay with indicated concentrations of HELQ and RPA (40 nM) in the presence of ATPγS (n=2). **h**, Representative gel of DNA annealing assay with indicated concentrations of HELQ(K365M) in the absence and presence of RPA (40 nM). **i**, Quantification of experiments such as shown in **h**. n = 4 independent experiments; mean ± S.E.M. **j**, Representative gel of DNA annealing assay with HELQ(K365M) (60 nM) and indicated concentrations of RPA. **k**, Quantification of experiments such as shown in j. n = 3 independent experiments; mean ± S.E.M. **l**, Representative gel of DNA annealing assay with HELQ (10 nM) and various concentrations of SSB. **m**, Quantification of experiments such as shown in l. n = 4 independent experiments; mean ± S.E.M.

As shown above, a strong reduction in unwound product was observed at higher concentrations of HELQ (Fig. 1a, b and Extended Data Fig. 1b–e); we reasoned that this could result from the reannealing of the unwound product. Indeed, reactions containing an unlabelled ‘cold’ oligonucleotide (oligo) yielded an increase in unwound product with excess HELQ (Extended Data Fig. 5a (compare lanes 3 and 4 with 7 and 8)). Kinetic analysis also showed that HELQ initially unwinds the substrate but then reanneals it back together at later time points (Extended Data Fig. 5b). Prompted by this, we directly tested HELQ for DNA strand annealing activity without and with an excess of RPA needed for 100% ssDNA coverage (theoretically, 16 nM RPA covers 10 nM ssDNA). We found that HELQ efficiently anneals complementary DNA strands either without or with RPA (Fig. 2a, b). At lower concentrations of HELQ, RPA stimulated HELQ DNA annealing activity by around twofold. However, at higher concentrations, HELQ showed greater DNA annealing activity in the absence of RPA. This raised the possibility that RPA aids HELQ loading on ssDNA when HELQ is present in limiting amounts. Titration experiments confirmed that substochiometric levels of RPA are sufficient to stimulate HELQ annealing activity (Fig. 2c, d). Notably, HELQ could still anneal complementary DNA strands in the presence of excess RPA (Extended Data Fig. 5c, d).

**Extended Data Fig. 6:**
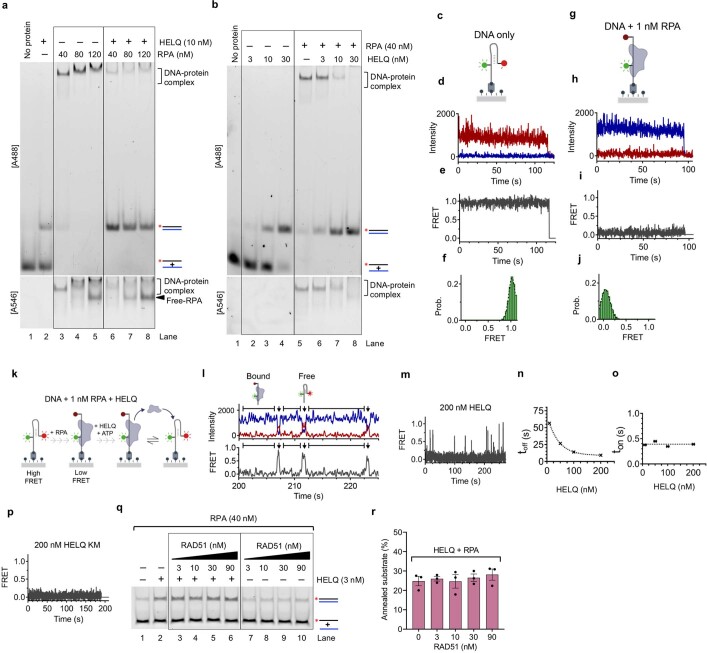
HELQ strips RPA from ssDNA. **a**. Native gel (longer 6% polyacrylamide) showing RPA stripping assay with HELQ (10 nM) alone and with indicated concentrations of RPA. **b**, Native gel (longer 6% polyacrylamide) showing RPA stripping assay with indicated concentrations of HELQ in the absence and presence of RPA (40 nM). **c**, Schematic of immobilized dual labelled (Cy3 and Cy5) DNA in the absence of RPA. **d**–**e**, Representative intensity trajectory (top) and corresponding FRET trajectory (bottom) of dual labelled DNA in the absence of RPA. **f**, Time-binned FRET histogram of DNA only, fit with gaussian. **g**, Schematic of immobilized dual labelled DNA in the presence of RPA. **h**–**i**, Representative intensity trajectory (top) and corresponding FRET trajectory (bottom) of dual labelled DNA in the presence of RPA. Stable high FRET is observed. **j**, Time-binned FRET histogram of DNA in the presence of RPA. Fit with gaussian. **k**, Schematic of the experimental set up of single-molecule FRET-based RPA striping assay. DNA dual-labelled with the FRET pair Cy3 and Cy5 is immobilized on the microscope slide. In the absence of RPA, a short 6 nt sequence of homology causes the DNA to fold into a high FRET state. Upon RPA binding, the DNA unfolds resulting in a low FRET state. Addition of HELQ results in cycling between the low (bound) and high (free) FRET states as RPA is bound and removed respectively. **l**, Example of single-molecule fluorescence trajectory (top, Cy3 in blue, Cy5 in red) and corresponding FRET (bottom) showing the transition from low FRET (bound) to high FRET (free). **m**, Representative FRET trajectory of DNA in the presence of 1 nM RPA and 200 nM HELQ, spikes of high FRET correspond to RPA removal events. **n**, Plot of dwell time of RPA bound (t_off_), low FRET, state with increasing HELQ concentration. **o**, Plot of dwell time of free state (t_on_), high FRET, on RPA removal with increasing HELQ concentration. **p**, Representative FRET trajectory of DNA in the presence of 1 nM RPA and 200 nM HELQ KM. **q**, Representative gel of DNA annealing assay with HELQ (3 nM), RPA (40 nM) and indicated concentrations of RAD51. **r**, Quantification of experiments such as shown in q. n = 3 independent experiments; mean ± S.E.M.

**Extended Data Fig. 7:**
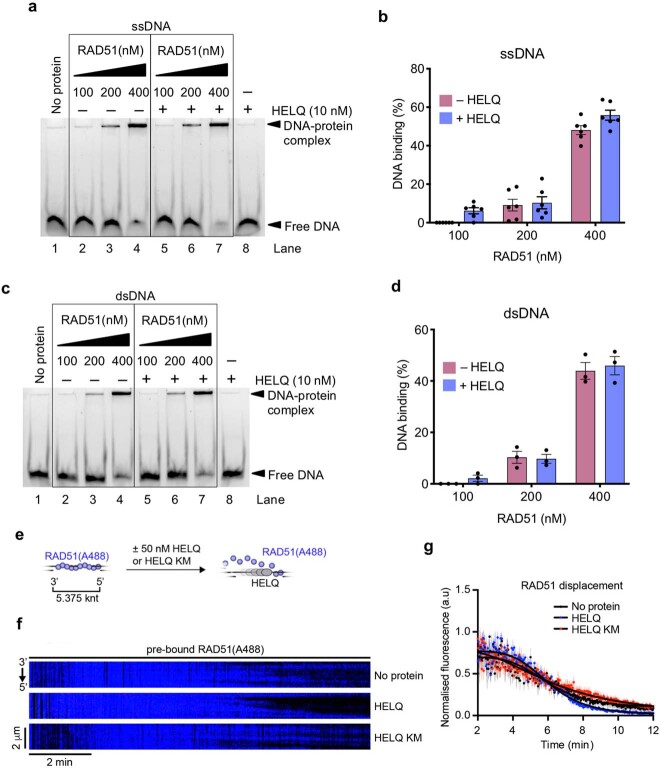
HELQ weakly displaces RAD51 from ssDNA. **a**, Representative EMSA gel showing RAD51 binding to ssDNA in the absence and presence of HELQ. Reactions were incubated at 37 °C for 20 min. **b**, Quantification of experiments such as shown in a. n = 6 independent experiments; mean ± S.E.M. **c**, Representative EMSA gel showing RAD51 binding to dsDNA in the absence and presence of HELQ. Reactions were incubated at 37 °C for 20 min. **d**, Quantification of experiments such as shown in c. n = 3 independent experiments; mean ± S.E.M. **e**, Schematic of the experimental set up of single-molecule FRET-based RAD51 removal assay. **f**, Representative kymographs of removal of Alx–RAD51 pre-bound to gapped DNA in the presence and the absence of 50 nM HELQ or HELQ(K365M). **g**, Removal of Alx–RAD51 measured from gapped ssDNA as a function of time in indicated conditions. Traces represent individual DNA molecules (n ≥ 3).

We next tested the requirement of ATP binding and hydrolysis for DNA annealing by HELQ. Surprisingly, in the presence of RPA, HELQ showed no DNA annealing without ATP, whereas ATP became dispensable when RPA was excluded from the reaction (Fig. 2e, f and Extended Data Fig. 5e, f). Even in the absence of RPA, ATP stimulated the DNA annealing activity of HELQ (Extended Data Fig. 5e, f). HELQ also failed completely to anneal DNA with ATPγS in the presence of RPA (Extended Data Fig. 5g). Collectively, these data suggest that HELQ possesses intrinsic DNA annealing activity that requires ATP binding and hydrolysis when ssDNA is coated with RPA. We next tested the helicase-inactive HELQ(K365M) mutant for DNA annealing activity and found that HELQ(K365M) is defective for DNA annealing in the presence of excess RPA but could anneal ssDNAs when RPA is excluded from reactions (Extended Data Fig. 5h, i). A titration experiment showed that, in contrast to the WT, HELQ(K365M) becomes progressively impaired by increasing concentrations of RPA (Fig. 2g, h and Extended Data Fig. 5j, k). RPA also failed to stimulate HELQ(K365M) (Fig. 2g, h). We also tested *E. coli* SSB protein and found that it only weakly stimulates HELQ annealing activity (Extended Data Fig. 5l, m). The N-terminal fragment of HELQ was previously shown to displace RPA from ssDNA^6^. However, full-length HELQ was not analysed for such activity. Thus, we directly tested RPA displacement from ssDNA during DNA strand annealing by omitting the deproteination step. We observed that HELQ can strip an excess of RPA from ssDNA, which occurred coincidently with the appearance of the annealed products (Extended Data Fig. 6a, b). To directly visualize RPA stripping from ssDNA by HELQ, we measured RPA–eGFP displacement by SMI analysis and found that WT HELQ (*k* = 0.136 ± 0.008 min^−1^) could efficiently strip RPA from ssDNA but HELQ(K365M) (*k* = 0.017 ± 0.004 min^−1^) could not (Fig. 2i–k). Using a single-molecule Förster resonance energy transfer (FRET)-based assay (Extended Data Fig. 6c–j), we observed concentration-dependant RPA stripping by WT HELQ, followed by rebinding of RPA (Extended Data Fig. 6k–n). The RPA rebinding is independent of HELQ concentration, indicating a constant transition rate from the free (*t*_on_) to bound (*t*_off_) state at various HELQ concentrations (Extended Data Fig. 6o). HELQ(K365M) did not show any RPA stripping (Extended Data Fig. 6p), indicating that active RPA stripping has a critical role in HELQ-mediated DNA annealing. Finally, we found that the addition of RAD51 had no effect on HELQ-dependent DNA annealing activity (Extended Data Fig. 6q, r). To study whether, like RPA, HELQ can also strip RAD51 from ssDNA, we directly tested RAD51 removal from ssDNA and dsDNA. HELQ did not remove RAD51 from either ssDNA or dsDNA in bulk assays (Extended Data Fig. 7a–d) and only weakly displaced RAD51 from ssDNA in our SMI setup (Extended Data Fig. 7e–g). This suggests that HELQ is unlikely to disrupt fully formed RAD51 nucleofilaments but might remove RAD51 that is bound to short-resected DNA, as is present during microhomology-mediated end joining (MMEJ).

## HELQ captures RPA–ssDNA

**Fig. 3:**
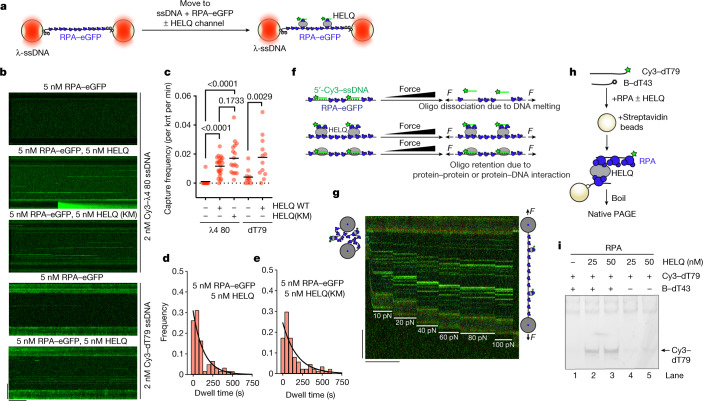
HELQ exhibits sequence-independent RPA-coated DNA capture activity. **a**, Schematics of the experimental optical tweezer set-up for observing the capture of Cy3-labelled DNA oligos. **b**, Kymographs showing the capture of targeted Cy3-labelled 80-mer oligo (λ4) in *trans* with HELQ and HELQ(K365M) in the presence of RPA–eGFP at multiple positions of RPA–eGFP-coated λ-ssDNA (top). KM, HELQ(K365M). Kymographs showing the capture of non-targeted Cy3-labelled dT 79 homopolymer in *trans* with HELQ in the presence of RPA–eGFP at multiple positions of RPA–eGFP-coated λ-ssDNA (bottom). Scale bars, 60 s (horizontal), 10 µm (vertical). **c**, Quantification of the experiments shown in **b**. Each datapoint represents a single DNA molecule. Data are mean ± s.d. Statistical analysis was performed using two-sided Mann–Whitney *U*-tests. No adjustments were made for multiple comparisons. **d**, The dwell times of captured λ4 by HELQ in the presence of RPA–eGFP. *n* = 61. The black line represents the exponential fit. Tau = 134 s. **e**, The dwell times of captured λ4 by HELQ(K365M) in the presence of RPA–eGFP. *n* = 87. The black line represents the exponential fit. Tau = 179 s. **f**, The experimental conditions for the optical tweezer experiment in **g**. **g**, Kymograph showing the Cy3–λ4 oligo captured on RPA–eGFP-coated λ-ssDNA in the presence of HELQ and RPA–eGFP after stretching of tethered λ-ssDNA by a gradual increase of force. Scale bars, 60 s (horizontal), 10 µm (vertical). **h**, Schematic of the bulk capture assay. **i**, Native gel showing the capture assay with the indicated concentrations of HELQ and RPA (82 nM). B indicates biotin at the 3′ end of B-dT43 oligo. The experiment was performed twice with similar results.

**Extended Data Fig. 8:**
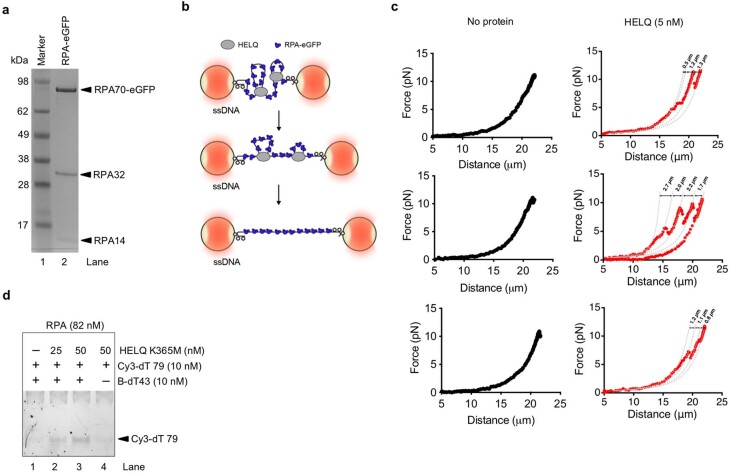
HELQ can capture non-complementary DNA strands in presence of RPA. **a**, SDS-PAGE gel (4-12%) showing purified recombinant human RPA–eGFP from *E. coli*. The gel was stained with CBB. Single preparation of RPA–eGFP used in this study. **b**, A schematic of DNA pulling process. RPA–eGFP-coated ss-λ DNA tethered between the two streptavidin beads (~4.8 μm diameter) was collapsed by bringing beads at <5 μm distance. Beads were subsequently pulled apart at constant speed (step size = 0.2 μm, frequency = 500 Hz). Force-extension curves were then recorded. **c**, Force-distance curves of individual eGFP-RPA-coated ss-λ DNA molecules recorded in the absence or presence of HELQ (left and right panels, respectively). Sawtooth-like patterns in the FD curves (red) indicate disruption of ssDNA loops held together by HELQ. The average loop sizes (1.5 ± 0.5 μm, N = 10) were estimated from the differences in the fitted contour lengths between the disruption events (dashed lines). **d**, Native gel showing the capture assay with the indicated concentrations of HELQ(K365M) in the presence of RPA (82 nM). The experiment performed two times with similar results.

RAD52 has been shown to possess robust DNA strand annealing activity and has a central role in single-strand annealing (SSA) repair of DSBs^10–14^. To investigate the mechanistic basis of the strand annealing ability of HELQ, we modelled our experiments on RAD52 annealing activity. Using optical tweezers, it was previously shown that RAD52 can *trans*-capture labelled oligos at multiple sites along 𝜆-DNA, independent of DNA sequence^15^. Using a similar set-up, we tested the ability of HELQ to capture a Cy3-labelled 80-mer oligo ssDNA in the presence of RPA–eGFP (around 100% coverage; Fig 3a and Extended Data Fig. 8a). HELQ facilitated the capture of 𝜆4 oligo^16,17^ at multiple sites (Fig. 3b, c). Notably, in contrast to its annealing or unwinding activity, HELQ(K365M) exhibited efficient DNA capture activity (Fig. 3b, c). We also analysed the dwell times of captured oligos and found that HELQ(K365M) showed moderately increased dwell times compared with the WT (Fig. 3d, e), most likely due to slightly higher DNA binding of HELQ(K365M). We also tested a 79-nucleotide T-homopolymer (dT79) and obtained similar capture frequencies as with the 𝜆4 oligo (Fig. 3c).

HELQ showed capture of both homologous (λ4) and T-homopolymer oligos at various sites on λ-DNA. To determine whether microhomologies present in ~50 kb λ-DNA could explain the capture pattern of both oligos, we performed forced-stretching experiments as shown in Fig. 3f. As DNA starts to melt at forces of >60 pN, we reasoned that, if HELQ oligo capture involves base-pairing interactions, short microhomologies should dissociate faster than the ones with longer homology. However, even at very high force (90–100 pN), all oligos remained engaged with ssDNA, irrespective of position (Fig. 3g). We next performed pulling experiments^18^ and observed characteristic force spikes when beads were pulled apart at a low force (10–15 pN) (Extended Data Fig. 8b, c). These spikes correspond to the disruption of HELQ complexes capturing RPA-coated ssλ-DNA in *cis*. To examine this further, we developed a bulk capture assay, in which we attempted to pull out labelled non-complementary DNA (Cy3–dT79) with a biotinylated dT43 oligo with HELQ. We found that both WT HELQ and the HELQ(K365M) mutant could capture non-complementary oligos bound to RPA (Fig. 3h, i and Extended Data Fig. 8d). Thus, HELQ and HELQ(K365M) can both capture DNA strands independent of sequence, probably through DNA tethering, but only the WT can anneal RPA-coated complementary DNA strands through RPA stripping. Interestingly, yeast Rad52, when bound to RPA-coated ssDNA clusters, can capture additional free RPA in pre-existing Rad52–RPA–ssDNA clusters^19^. This activity was postulated to be important for second-end capture during homologous recombination (HR).

## HELQ functions in SSA and MMEJ

**Fig. 4:**
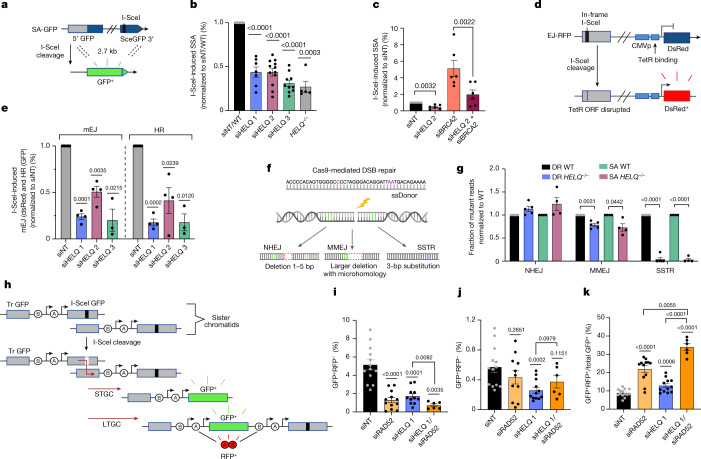
HELQ functions in DSB repair, SSA and MMEJ. **a**, Schematic of the SA-GFP reporter assay. **b**, I-SceI-induced SSA frequency in U2OS-SA *HELQ*^*–/–*^ cells and WT cells treated with the indicated short interfering RNA (siRNA). *n* = 17 (non-targeting siRNA (siNT)/WT), *n* = 7 (siHELQ 1), *n* = 12 (siHELQ_2), *n* = 10 (siHELQ 3) and n = 5 (*HELQ*^*–/–*^) independent experiments. Data are mean ± s.e.m. **c**, SSA frequency in U2OS-SA cells treated with the indicated siRNA. *n* = 6 independent experiments. Data are mean ± s.e.m. **d**, Schematic of the EJ-RFP reporter assay. **e**, I-SceI-induced mutagenic end-joining (mEJ-RFP) and HR (DR-GFP) frequencies in U2OS-EJDR cells treated with the indicated siRNA. *n* = 7 (siNT), *n* = 4 (siHELQ 1), *n* = 2 (siHELQ 2) and *n* = 3 (siHELQ 3) independent experiments. Data are mean ± s.e.m. **f**, Schematic of Cas9-mediated DSB repair assay. **g**, Cas9-mediated DSB repair assay showing the frequencies of NHEJ, MMEJ and SSTR repair in *HELQ*^–/–^ and WT U2OS-DR or U2OS-SA cells. *n* = 5 (U2OS-DR WT and DR *HELQ*^*–/–*^) and *n* = 4 (U2OS-SA and SA *HELQ*^*–/–*^) independent experiments. Data are mean ± s.e.m. **h**, Schematic of the RFP-SCR reporter assay. Tr GFP, truncated GFP. **i**, I-SceI-induced STGC frequency in U2OS-RFP-SCR cells treated with the indicated siRNA. *n* = 13 (siNT) *n* = 11 (siHELQ 1), *n* = 12 (siRAD52) and *n* = 6 (siHELQ 1/siRAD52) independent experiments. Data are mean ± s.e.m. **j**, LTGC frequency as shown in **i**. *n* = 13 (siNT), *n* = 12 (siHELQ 1), *n* = 11 (siRAD52) and *n* = 6 (siHELQ 1/siRAD52) independent experiments. Data are mean ± s.e.m. **k**, The ratio of LTGC/total GC from the experiments in **i** and **j**. *n* = 14 (siNT), *n *= 12 (siHELQ 1), *n* = 12 (siRAD52) and *n* = 6 (siHELQ 1/siRAD52) independent experiments. Data are mean ± s.e.m. For **b**, **c**, **e**, **g**, **i**–**k**, statistical analysis was performed using two-tailed paired *t*-tests as compared with siNT, unless otherwise indicated.

**Extended Data Fig. 9:**
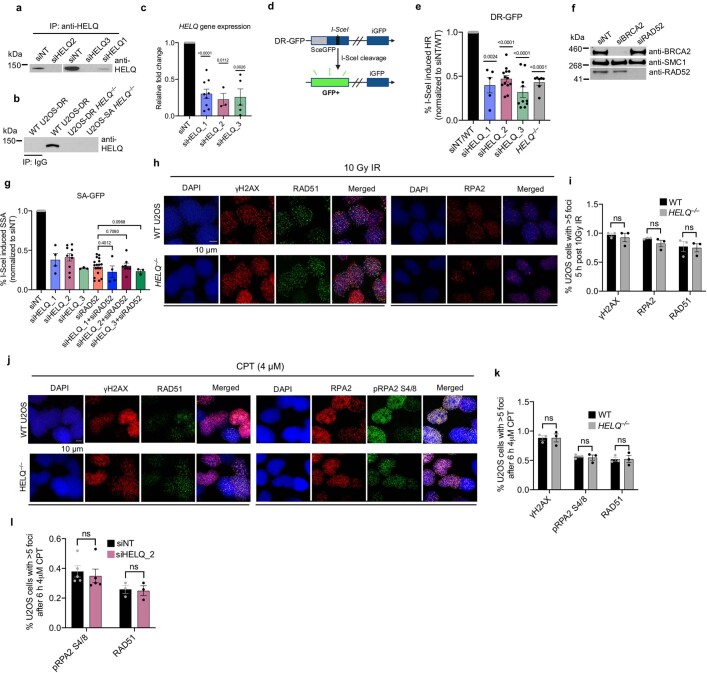
HELQ is epistatic with RAD52 for SSA and additive for second-end capture. **a**, HELQ protein levels in U2OS WT cells, post 72 h post siRNA transfection, confirmed by HELQ-immunoprecipitation and western blot. **b**, HELQ protein levels in U2OS WT and *HELQ*^*–/–*^ cells, confirmed by HELQ-immunoprecipitation and western blot. **c**, *HELQ* gene expression after treatment with siRNA post 72 h in U2OS cells. siNT n = 9, siHELQ_1 n = 9, siHELQ_2 n = 3, siHELQ_3 n = 3 independent experiments ± S.E.M, compared to siNT. **d**, Schematic representation of DR-GFP reporter assay for measuring DSB repair by HR. **e**, I-SceI-induced HR frequency in U2OS DR *HELQ*^*–/–*^ cells and in U2OS-DR cells treated with the indicated siRNA. siNT/WT, n = 20; siHELQ_1, n = 5; siHELQ_2, n = 13; siHELQ_3, n = 10; *HELQ*^*–/–*^, n = 7 independent experiments ± S.E.M, compared to siNT or WT cells. **f**, BRCA2 and RAD52 proteins levels 72 h post siRNA transfection confirmed by western blot. **g**, I-SceI-induced SSA frequency in U2OS-SA cells treated with the indicated siRNA. siNT, n = 21; siRAD52, n = 4; siHELQ_1, n =4; siHELQ_2, n = 10; siHELQ_3, n = 3; siHELQ_1/RAD52, n = 4, siHELQ_2/RAD52, n = 8; siHELQ_3/RAD52, n = 3 independent experiments ± S.E.M, compared to siNT. **h**, Detection of formation of γH2AX, RPA2, and RAD51 foci after 10 Gy IR treatment in U2OS WT and *HELQ*^*–/–*^ cells. **i**, Quantification of RPA2, RAD51 and γH2AX foci in experiments as shown in h. n = 3 independent experiments ± S.E.M, compared to siNT. **j**, Detection of formation of γH2AX, RPA2, pRPA2 S4/8 and RAD51 foci after treatment with 4 µM CPT (Camptothecin) of U2OS WT and *HELQ*^*–/–*^ cells. **k**, Quantification of γH2AX, pRPA S4/8 foci and RAD51 foci in experiments as shown in j. n = 3 independent experiments ± S.E.M, compared to siNT. **l**, Quantification of formation of pRPA2 S4/8 and RAD51 foci after 6 h CPT treatment in U2OS cells treated with indicated siRNA. pRPA2 S4/8 n = 5, RAD51 n =3 independent experiments ± S.E.M, compared to siNT. siNT, siRNA not-targeted. The statistical significance was determined by using two-tailed paired t test.

To extend our findings with HELQ to DSB repair in vivo, we first confirmed that deletion of *HELQ* or protein depletion in cells inhibits HR (Extended Data Fig. 9a–e). As DNA annealing is required for SSA repair, we investigated a potential role for HELQ in this process. Strikingly, HELQ depletion or *HELQ* deletion impaired SSA repair of an integrated SSA reporter (SA-GFP; Fig. 4a, b). Although depletion of the HR factor, BRCA2, increases SSA repair, this was strongly reduced by HELQ depletion (Fig. 4c and Extended Data Fig. 9f). Consistent with an epistatic role in SSA, co-depletion of RAD52 and HELQ did not further decrease SSA repair compared with individual depletions (Extended Data Fig. 9g). We also assessed whether HELQ functions in alternative end joining repair, which also involves an annealing step. Using cells containing both EJ-RFP and DR-GFP reporter systems for simultaneous detection of alternative end joining and HR, respectively, we observed a significant reduction in both DSB repair pathways after HELQ depletion (Fig.4d, e). Alternative end joining encompasses broad non-nonhomologous end joining (NHEJ) repair events including MMEJ. To specifically study the role of HELQ in MMEJ, we used a Cas9-mediated DSB repair assay in which the fate of DSB repair by MMEJ, NHEJ or single-stranded templated repair (SSTR) can be determined^20^ (Fig. 4f). The loss of *HELQ* resulted in a slight increase in NHEJ, whereas MMEJ was significantly impaired and SSTR was completely abolished (Fig. 4g). Interestingly, DNA annealing is important for SSTR^21,22^. A role for HELQ in DNA end resection could potentially explain defects in HR, SSA and MMEJ. However, we did not observe any reduction in RPA or RAD51 (refs. ^3,23,24^) focus formation after irradiation or camptothecin treatment in *HELQ*^–/–^ or depleted cells, excluding a role for HELQ in resection (Extended Data Fig. 9h–l). Impaired DNA strand annealing during second-end capture in DSB repair or failure to capture the repaired strand in synthesis-dependent strand annealing can result in a shift towards long-tract gene conversion (LTGC)^25–29^. Using the same reporter system, we found that, similar to RAD52, HELQ deficiency results in a strong and moderate reduction in short-tract gene conversion (STGC) and LTGC, respectively, with the LTGC/total gene conversion (GC) ratio showing a significant bias towards LTGC (Fig. 4h–k). Finally, co-depletion of HELQ with RAD52 showed a further bias towards LTGC, implying that they have roles in parallel pathways during GC (Fig. 4k).

**Extended Data Fig. 10:**
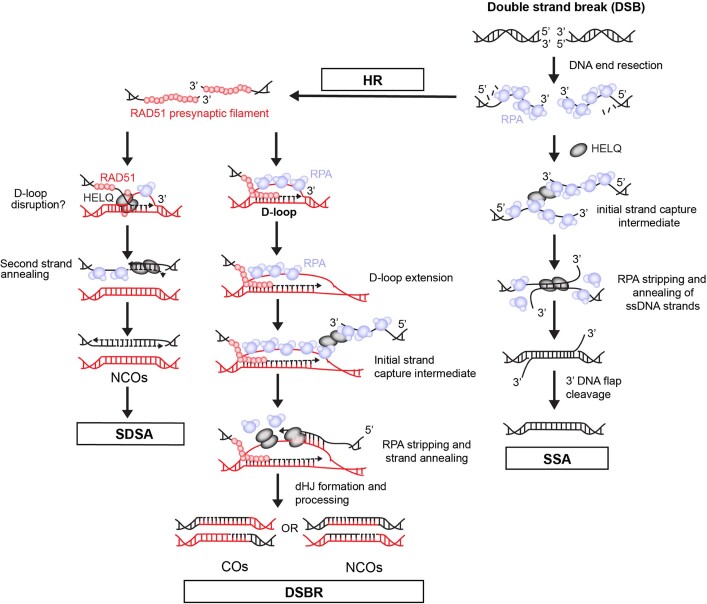
A possible mechanism of HELQ function in HR and SSA. **HR**, In HR, following DSB, 5′ DNA ends are resected by nucleases, which generates RPA-coated 3′ overhangs. RPA is displaced by RAD51 giving rise to presynaptic nucleoprotein filament formation. This RAD51-filament invades homologous DNA duplex through its 3′ end. DNA invasion result in D-loop formation, which is extended by DNA synthesis. If the SDSA pathway is invoked, HELQ can disrupt the D-loop to displace and/or anneal the extended strand with the broken duplex via its helicase and strand annealing activities. Alternatively, in DSBR, the D-loop can be greatly extended and RPA-coated DNA strands in D-loop and second 3′ overhang can be captured by HELQ. During this second-end capture step, HELQ strips RPA from ssDNA and anneals complementary DNA strands together to prime DNA synthesis, restoring the broken DNA and resulting in either crossover or non-crossover products. **SSA**, similar to HR, longer RPA-coated 3′ overhangs can be captured by HELQ. Post capture, HELQ strips RPA from both ssDNAs and actively anneals the DNA strands together using base-pairing. The remaining flaps are cleaved, and nicks are ligated by specific nuclease and ligases completing the DSB repair reaction.

In summary, our study implicates HELQ in several distinct DSB repair pathways, including HR, SSA and MMEJ, casting light on its role in genome stability and tumour avoidance. As these repair pathways each require DNA annealing steps, we propose that HELQ functions in these pathways through its intrinsic ability to capture RPA-bound ssDNA and then displace RPA to facilitate annealing of complementary DNA strands. HELQ alone or in complex with RAD51 might unwind D-loops before annealing complementary strands (Extended Data Fig. 10). The bias towards LTGC events after HELQ depletion is consistent with a role for DNA annealing by HELQ during second-strand capture and/or synthesis-dependent strand annealing during HR, which may explain why HR is reduced in HELQ-deficient cells. Finally, our finding that HELQ is epistatic with RAD52 for SSA but additive for GC during HR is surprising, as this implicates two DNA strand annealing enzymes in several distinct DSB repair processes in cells.

## Methods

### Preparation of expression vectors

Custom HELQ ORF was purchased from GeneArt and used as a template during PCR to prepare plasmid (pFastbac1) compatible for bacmid preparation for expression in insect cells. To prepare the MBP-HELQ-Flag construct, HELQ was amplified by PCR using the primers HELQ_F and HELQ_FLAG_R. The amplified insert was digested with BamHI and XmaI and inserted into the pFastbac1 vector containing MBP (maltose binding protein tag; previously inserted using the EcoRV and HindIII restrictions sites). The resulting construct was pFB-MBP-HELQ-FLAG. To prepare helicase-dead HELQ(K365M), pFB-MBP-HELQ-Flag was mutagenized with the primers HELQ_K365M_F and HELQ_K365M_R using the Q5 site-directed mutagenesis kit according to the manufacturer’s instructions. The constructs for pET11c-RAD51 and, RPA–eGFP (pMM801) and RPA-mRFP1 (pMM802) were gifts from L. Krejci and M. Modesti, respectively.

### Recombinant protein purification

To express proteins in insect cells, bacmid, primary and secondary baculoviruses were prepared according to the manufacturer’s instructions (Bac-to-bac system, Life technologies). To express recombinant MBP-HELQ-FLAG, *Spodoptera frugiperda* (Sf9) insect cells were seeded at 500,000 cells per ml and, after around 24 h, cells were infected with MBP-HELQ-Flag baculovirus. The infected cells were incubated at 27 °C for 56 h with continuous agitation. Cells were collected by centrifugation at 500*g* for 10 min and washed once with 1× PBS (137 mM NaCl, 2.7 mM KCl, 10 mM Na_2_HPO_4_, 1.8 mM KH_2_PO_4_). The collected pellets were snap-frozen in liquid nitrogen and stored at −80 °C until further use. All subsequent steps were performed either on ice or at 4 °C. The cells pellets were resuspended in 3 volumes of lysis buffer containing 50 mM Tris-HCl pH 7.5, 1 mM ethylenediaminetetraacetic acid (EDTA), protease inhibitor cocktail tablets (Roche), 30 µg ml^−1^ leupeptin (Merck), 1 mM phenylmethylsulfonyl fluoride (PMSF), 1 mM dithiothreitol (DTT), 0.1% NP-40 substitute (NP-40) and incubated for 15 min with continuous agitation. Next, 50% glycerol and 5 M NaCl were added sequentially to the final concentrations of 16.7% and 310 mM, respectively, and the suspension was further incubated for 30 min with continuous agitation. The suspension was centrifuged at ~48,000*g* for 30 min to obtain the soluble extract. The amylose resin (NEB) was pre-equilibrated with amylose wash buffer I (50 mM Tris-HCl pH 7.5, 1 mM 2-mercaptoethanol (β-ME), 1 M NaCl, 1 mM PMSF, 10% glycerol and 0.1% NP-40) and added to 50 ml tubes containing soluble extract. These tubes were subsequently incubated for 1 h with continuous rotation. After incubation, the resin was washed batchwise four times by centrifugation at 2,000*g* for two min and twice on column with amylose wash buffer I. Resin was washed twice more on column with amylose wash buffer II (same as wash buffer I but with 0.5 mM β-ME and 0.8 M NaCl). Protein was eluted from the resin using amylose elution buffer (same as amylose wash buffer II supplemented with 10 mM maltose) and total protein was estimated using the Bradford assay. To remove the MBP tag, 1/8 (w/w) of PreScission protease to the total protein was added to amylose eluate and incubated for 2 h without rotation but with gentle agitation at regular intervals. The Flag resin (anti-Flag M2 resin, Sigma-Aldrich), which was pre-equilibrated with Flag wash buffer (50 mM Tris-HCl pH 7.5, 0.8 M NaCl, 1 mM PMSF,10% glycerol), was added to amylose eluate containing PreScission protease and incubated for 2 h with continuous rotation. Flag resin was collected directly on column and washed six times with Flag wash buffer. The protein was eluted from Flag resin with Flag elution buffer (50 mM Tris-HCl pH 7.5, 0.5 mM β-ME, 150 mM NaCl, 1 mM PMSF,10% glycerol, 150 ng µl^−1^ 3×Flag peptide (Sigma-Aldrich)), aliquoted, frozen in liquid nitrogen and stored at −80 °C. The same purification procedure was used to purify HELQ(K365M).

Recombinant human RAD51 was purified as described previously with a few modifications^30^. The pET11c-RAD51 expression vector was transformed into *E. coli* BLR(DE3)pLysS cells and subsequent culture containing ampicillin (100 mg l^−1^) and chloramphenicol (33 mg l^−1^) was grown to an optical density (OD) at 600 nm of 0.7. RAD51 expression was induced with 1 mM isopropyl β-d-1-thiogalactopyranoside (IPTG) at 37 °C for 3–4 h. All of the subsequent steps were performed either on ice or at 4 °C. Cells were collected by centrifugation at 5,000*g*. Cell pellets were resuspended in cell breakage buffer (50 mM Tris-HCl pH 7.5, 10% sucrose, 0.5 mM EDTA, 1 M KCl, 1 tablet per 50 ml of protease inhibitor cocktail tablets (Roche), 1 mM PMSF, 1 mM DTT and 0.01% NP-40), sonicated and centrifuged at 100,000*g* for 1 h. To precipitate RAD51, 0.242 g ml^−1^ ammonium sulphate was mixed with clarified supernatant and centrifuged for 20 min at 10,000*g*. The pellet was resuspended with buffer K (20 mM K_2_HPO_4_ pH 7.5, 10% glycerol, 0.5 mM EDTA, 1 mM DTT, 0.01% NP-40) and loaded onto the Q Sepharose Fast flow column (Cytiva), pre-equilibrated with K buffer-low (K buffer supplemented with 175 mM KCl). The column was washed extensively with K buffer-low and protein was subsequently eluted with a KCl gradient using K buffer-high (K buffer supplemented with 0.6 M KCl). The eluted fractions containing RAD51 were pooled and diluted with 6 volumes of dilution buffer (25 mM Tris HCl pH 7.5, 0.5 mM EDTA, 1 mM DTT, 0.01% NP-40). The diluted sample was loaded onto the HiTrap Heparin HP affinity column (Cytiva), which was pre-equilibrated with buffer H without glycerol (25 mM Tris HCl pH 7.5, 0.5 mM EDTA, 1 mM DTT, 0.01% NP-40, 150 mM KCl) and washed with buffer H containing 10% glycerol. Protein was eluted in buffer H with a KCl gradient (0.1 M to 1 M KCl) and the fractions containing RAD51 were pooled and dialysed in buffer H without glycerol. The dialysed sample was loaded onto the Mono Q 5/50 GL column (Cytiva), equilibrated with buffer Q (25 mM Tris HCl pH 7.5, 0.5 mM EDTA, 1 mM DTT, 0.01% NP-40, 100 mM KCl, 10% glycerol) and the column was further washed with buffer Q containing 50 mM KCl but lacking glycerol. RAD51 was eluted from the Mono Q column with a KCl gradient (0.05 M to 1 M) in buffer Q lacking glycerol. The eluted fractions containing RAD51 were pooled and further concentrated with the Vivaspin Centrifugal Concentrator (30 kDa molecular weight cut-off (MWCO)). Glycerol was added to the concentrated sample to a final concentration of 10%. Finally, the samples were aliquoted, frozen in liquid nitrogen and stored at −80 °C. The RAD51(C319S) mutant was purified using same procedure. RPA–mRFP1 and RPA–eGFP were purified as described previously^31^. Recombinant RecA (M0249) and ET SSB (M2401) were commercially purchased from NEB, England.

To purify GST and GST–BRC4, the BRC4 construct was cloned into the pGEX6P-1 vector containing a GST tag using BamHI and EcoRI restriction sites. The pGEX6P-1 and pGEX6P-1–BRC4 constructs were transformed into BL21 DE3 cells and plated onto an agar plate containing ampicillin (100 µg ml^−1^). A single colony was isolated and inoculated into 6 ml preculture overnight. The next day, 4.5 ml preculture medium was added to 450 ml LB medium containing ampicillin (100 µg ml^−1^), and the OD at 600nm was monitored at regular intervals. Proteins were induced with 1 mM IPTG at 0.6 OD and cultures were incubated for 4 h. The cell pellets were collected, washed with cold PBS and stored at −80 °C. For purification, the cell pellets were lysed by sonication in PBS and the samples were centrifuged at 75,600*g* for 30 min at 4 °C. Next, the supernatant was collected and incubated with 1.2 ml glutathione resin for 1 h at 4 °C. The resin was washed three times with PBS and the proteins were eluted with elution buffer (20 mM Tris-HCl pH 7.5, 20 mM glutathione). The eluted proteins were aliquoted, snap-frozen in liquid nitrogen and stored at −80 °C.

### Preparation of labelled proteins

The RAD51(C319S) variant was expressed and purified as described earlier for WT RAD51 (refs. ^30,32^). After purification, the protein was fluorescently labelled with Alexa Fluor 488 C_5_ maleimide (Thermo Fisher Scientific, A10254) according to previously described protocol^31^. Labelled protein was purified away from the free dye using the Zeba column gel filtration system (0.5 ml resin, 50 kDa MWCO). The protein concentration was estimated by Coomassie staining and dye concentration was measured spectrophotometrically. The presence of minimum free dye concentration was assessed using SDS–PAGE on labelled proteins. The protein to dye concentration ratio was consistently 0.9–1.0. D-loop formation of labelled RAD51 was tested and gave yields comparable to unlabelled WT RAD51 protein, consistent with previous reports^32^. RPA–eGFP and RPA–mRFP1 were expressed and purified as described previously^31^. DNA binding of labelled RPA was tested. All RPA-fusion proteins displayed similar ssDNA affinities within nanomolar *K*_d_ range.

### Preparation of DNA substrates and oligonucleotides used for in vitro analysis

All DNA oligonucleotides used in the in vitro analysis were commercially synthesized and purchased from Merck Life Sciences. To prepare various substrates used in this study, when needed, combination(s) of DNA oligonucleotides were annealed together by mixing and heating them at 95 °C for 3 min, followed by gradual cooling of the samples overnight. The names and sequences of oligos used were as follow: oligo 1 (5′ FITC-AGCTACCATGCCTGC ACGAATTAAGCAATTCGTA ATCATGGTCATAGCT), oligo 2 (5′-AGCTATGACCATGATT ACGAATTGCTTAATTCGTGCAGGCATGGTAGCT, oligo 4 (AATTCGTGCAGGCATGGTAGCT), oligo 5 (AGCTATGACCATG ATTACGAATTGCTT), oligo 6 (AGCTATGACCATGATTACGAATTGCTTGGAATCCTGACGAACTGTAG), oligo 23 (5′-FITC-GACGCTGCCGAATTCTACCAG TGCCTTGCTAGGACATCTTTGCC CACCTGCAGGTTCACCC), oligo 22 (GGGTGAACCTGCAGGTGGG CG GCTGCTCATCGTAGGTTAGTTGGTAGAATTCGGCAGCGTC), oligo 21 (TAAGAGCAAGATGTTCTATAAAA GATGTCCTAGCAAGGCAC), oligo 20 (TATAGAACATCTTGCTCTTA); oligo F (5′-FAM-AGCTACCATGCCTGCACG AATTAAGCAATTCGTAA TCATGGTCATAGCT) and oligo R (AATTCGTGCAGGCATGGTAGCT-ROX-3′**)**. FITC, FAM and ROX indicate the position of fluorescein isothiocyanate, 6-carboxyfluorescein and rhodamine, respectively in oligos above. The combinations of oligos were annealed together to prepare 3′ overhang (oligo 1 + oligo 4), 5′ overhang (oligo 1 + oligo 5), dsDNA (oligo 1 + oligo 2), Y structure (oligo 1 + oligo 6), lagging strand fork (oligo 1 + oligo 4 + oligo 6), D-loop (oligo 23 + oligo 22, oligo 21 + oligo 20) and 3′ overhang used for quenching kinetic assay (oligo F + oligo R). Oligo 1 was used as the ssDNA substrate. The additional oligos used were as follows; λ4 (5′-Cy3-CCTGAACGACCAG GCGTCTTCGTTCATC TATCGGATCGCCACACTCA CAACAATGAGTGGCAGATAT AGCCTGGTGGTTC), dT79 (5′-Cy3-TTTTTTTTTTTTTTTTTTTTTTTTTTTTTTTTTTTTTTTTTTTTTTTTTTTTTTTTTTTTTTTTTTTTTTTTTTTTTTT) and B-dT43 (TTTTTTTTTTTTTTTTTTTTTTTTTTTTTTTTTTTTTTTTTTT-3′-Bio), where Bio indicates the position of biotin in the oligo sequence.

### DNA unwinding assay

The unwinding assays were performed in 15 µl helicase buffer containing 25 mM Tris-HCl pH 8.0, 2 mM ATP, 2 mM MgCl_2_, 1 mM DTT, 50 mM NaCl, 0.1 mg ml^−1^ bovine serum albumin (BSA, New England Biolabs), 1 mM PEP (phosphoenolpyruvate, Sigma-Aldrich), 10 U ml^−1^ pyruvate kinase (Sigma-Aldrich) and 5′-end-FITC-labelled 25 nM DNA substrate (in molecules). All of the steps except the assembling reactions and protein addition were performed in the dark. The reactions were assembled on ice and recombinant proteins were added, mixed and incubated at 37 °C for 30 min. The reactions were stopped with 5 µl of 2% stop solution (0.2% SDS, 30% glycerol, 150 mM EDTA, bromophenol blue) and 1 µl proteinase K (Roche, 18.4 mg ml^−1^) and incubated for 10 min at 37 °C. To prevent reannealing, 2% stop solution was supplemented with tenfold excess of unlabelled oligos with the same sequence as the FITC-labelled oligo. The products were resolved by 10% native polyacrylamide gel (19:1 acrylamide-bisacrylamide, Bio-Rad) using Mini-Protean Tetra Cell electrophoresis system (Bio-Rad) at 100 V for 1 h. The gels were directly imaged in ChemiDoc MP imaging system.

### Quenching-based kinetic assay for DNA unwinding

These assays were performed in 60 µl helicase buffer with 20 nM DNA substrate (in molecules). The oligo F (49-mer) in DNA substrate was labelled with 6-flouroscein amidite (6-FAM) at the 5′ end whereas oligo R (22-mer) was labelled at 3′ end with rhodamine. The reactions were assembled on ice in 96-microwell plate and the recombinant proteins were directly added to their respective wells. The microplate was transferred to a microplate reader (CLARIOstar, BMG Labtech) at 37 °C and 6-FAM intensity was continuously monitored at every 30 s for 60 min. The final values were plotted as graphs using GraphPad PRISM.

### Electrophoretic mobility shift assay

EMSA reactions (15 µl) were performed in binding buffer containing 25 mM Tris-HCl pH 8.0, 2 mM ATP, 2 mM MgCl_2_, 1 mM DTT, 50 mM NaCl, 0.1 mg ml^−1^ BSA (New England Biolabs) and 5′-end-FITC-labelled 25 nM DNA substrate (in molecules). All of the steps except for the assembling reactions and protein addition were performed in the dark. The reactions were assembled on ice and recombinant proteins were added to reactions, mixed and incubated for 10 min at 37 °C in the dark. Reactions were supplemented with 5 µl of EMSA loading buffer (50% glycerol, bromophenol blue) and resolved with 6% native TBE polyacrylamide gel (19:1 acrylamide-bisacrylamide, Bio-Rad) using the Mini-Protean Tetra Cell electrophoresis system (Bio-Rad) at 80 V for 45 min on ice. Finally, gels were imaged using the ChemiDoc MP imaging system. Scans of the gels are provided in the Supplementary Information.

### RPA stripping gel-based assay

The stripping assay was performed as described for EMSA except that the products were resolved at room temperature and longer 6% TBE native gel was used.

### DNA annealing assay

DNA annealing assays were performed in 15 µl annealing buffer containing 25 mM Tris-HCl pH 8.0, 2 mM ATP, 2 mM MgCl_2_, 1 mM DTT, 50 mM NaCl and 0.1 mg ml^−1^ BSA (New England Biolabs). All of the steps except for the assembling reactions and protein addition were performed in the dark. For DNA substrate, 10 nM (in molecules) complementary oligos (5′-FITC-oligo 1 and oligo 2) were separately incubated in 7.5 µl annealing buffer and as indicated, with or without RPA on ice for 2 min. Recombinant proteins were added to FITC-oligo 1 reactions (7.5 µl) on ice, immediately followed by the addition of oligo 2 reactions (7.5 µl). Reactions were incubated for 10 min at 37 °C. The final concentration of both individual oligo and annealed dsDNA product was 5 nM. The reactions were stopped with 5 µl of 2% stop solution (0.2% SDS, 30% glycerol, 150 mM EDTA, bromophenol blue) and 1 µl proteinase K (Roche, 18.4 mg ml^−1^) and incubated for 10 min at 37 °C. To prevent the detection of spontaneous annealing during deproteination, 25-fold excess of unlabelled oligo 1 to FITC-oligo 1 was included in the 2% stop solution. The products were resolved and imaged identically as described for the unwinding assays.

### Interaction assay

To study the interactions between HELQ and RAD51, MBP-HELQ-Flag baculovirus was expressed in 300 ml insect cells, and soluble extract from the collected pellet was prepared as described for the protein purification procedure. Reagent volumes for the preparation of the soluble extract were scaled down accordingly. The soluble extract was divided equally into two parts and incubated with amylose (E8021, NEB) and anti-Flag M2 resin (A2220, Sigma-Aldrich) for 1 h at 4 °C. Next, amylose resin and anti-Flag M2 resin were washed with wash buffer I (50 mM Tris-HCl pH 8.0, 1 mM DTT, 310 mM NaCl, 10% glycerol, 1 mM PMSF). Both resins were divided into 50 µl volumes in separates microtubes. 4 µg recombinant RAD51 was added to all except for one tube for each resin and incubated for 1 h at 4 °C. Resins were washed with wash buffer II (the same as wash buffer I but containing 100 mM NaCl). Proteins were eluted from resin in 1× SDS buffer by boiling at 95 °C for 4 min. The eluate was separated by 4–12% native SDS–PAGE gel (NuPAGE Bis-Tris, Invitrogen) and stained with instant blue Coomassie protein stain (Abcam).

### DNA capture assay

The capture assays were performed in 20 µl DNA annealing buffer supplemented with 0.05% Tween-20. The reactions were assembled on ice and, where indicated, 82 nM RPA, 10 nM biotinylated dT43 (bio-dT43) and 10 nM 3′Cy3–dT79 were added to reactions. Next, HELQ was added to reactions as indicated. Reactions were mixed and incubated at 37 °C for 8 min in the dark. To pull-down bio–dT43, magnetic streptavidin beads were washed twice with PBS-0.1% Tween-20 (Dynabeads M-280, Thermo Fisher Scientific) and 5 µl of beads was added to each reaction. Reactions were further incubated for 4 min in the dark at room temperature and then washed twice with 80 µl washing buffer (25 mM Tris-HCl pH 8.0, 2 mM ATP, 2 mM MgCl_2_, 1 mM DTT, 100 mM NaCl, 0.5 mg ml^−1^ bovine serum albumin, NP-40) on a magnetic rack. Finally, the beads were resuspended in 30 µl loading buffer (7.5 µl 2% stop solution and 22.5 µl washing buffer) and boiled at 95 °C for 4 min. The samples were centrifuged at high speed for 1 min and 25 µl volume sample was loaded immediately on 10% native polyacrylamide gel and run as described for the unwinding assay. The gels were directly imaged in the ChemiDoc MP imaging system (Bio-Rad).

### Substrate and flow cell preparation for SMI analysis

Experiments were performed using the commercially available C-trap (LUMICKS) set-up. Protein channels of the microfluidics chip were first passivated with BSA (0.1% (w/v) in PBS) and Pluronics F128 (0.5% (w/v) in PBS), with a minimum 500 µl of both flowed through before use. Biotinylated ssDNA precursor was prepared as described previously^33^. To generate gapped λDNA, a protocol described previously was used^9^. In brief, biotinylated hairpin oligonucleotides were annealed to λ-dsDNA ends and ligated^34^. *Streptococcus pyogenes* Cas9 D10A nickase (IDT) bound to previously described^16^ guide RNAs was subsequently used to generate targeted DNA nicks. The reaction was then stored at 4 °C and directly diluted in PBS on the day of the experiment. DNA was captured between 4.5 μm SPHERO Streptavidin-coated polystyrene beads at 0.005% (w/v) using the laminar flow cell, stretched and held at forces of 100 pN (for ssDNA) or 65 pN (λ-gDNA 4/5) until the strands were fully melted. The presence of ssDNA and/or a ssDNA gap was verified by comparison with a built-in freely joined chain model. For confocal imaging, three excitation wavelengths were used—488 nm for eGFP and Alexa Fluor 488, 532 nm for Cy3 and 638 nm for Cy5, with emission detected in three channels with blue filter 512/25 nm, green filter 585/75 nm and red filter 640 LP.

### Single-molecule DNA unwinding assay

For all the unwinding assays, the λ-gDNA 4/5 construct was held at a constant force of 50 pN. Beads and DNA were kept in PBS during the experiment, while DNA was melted in 0.5× NTM buffer (25 mM Tris-HCl pH 7.5, 50 mM NaCl, 0.5 mM MgCl_2_) supplemented with 0.2 mg ml^−1^ BSA and 1 mM DTT. HELQ (50 nM) and/or 25 nM RAD51(A488) were flowed into the system in 1× HELQ buffer (25 mM Tris-HCl pH 8.0, 2 mM MgCl_2_, 50 mM NaCl) supplemented with 2 mM ATP, 0.2 mg ml^−1^ BSA and 1 mM DTT. Unwinding was monitored by the change in the distance between the beads over time. To directly image fluorescent RAD51, the following image acquisition set-up was used: 4 µW blue laser power, 0.1 ms px^−1^ dwell time, 100 nm pixel size, 1 s interframe wait time.

### SMI-based RPA/RAD51 stripping

Using optical tweezers in the stripping assays, the λ-gDNA 4/5 construct was held at a distance corresponding to a force of 10 pN after melting. The beads and DNA were kept in PBS during the experiment (microfluidic channels 1 and 2), while DNA was melted in PBS (microfluidic channel 2) and subsequently incubated with 5 nM RPA–eGFP or a mixture of 100 nM RAD51 and 100 nM Alx–RAD51 in 1× HELQ buffer (25 mM Tris-HCl pH 8.0, 2 mM MgCl_2_, 50 mM NaCl), 0.2 mg ml^−1^ BSA and 1 mM DTT in channel 3. Once RPA–eGFP or Alx–RAD51 were assembled on λ-gDNA 4/5 (after ~2 min of incubation) beads with DNA were moved to channel 4 containing 50 nM HELQ in 1× HELQ buffer (25 mM Tris-HCl pH 8.0, 2 mM MgCl_2_, 50 mM NaCl) supplemented with 2 mM ATP, 0.2 mg ml^−1^ BSA and 1 mM DTT. RPA–eGFP or Alx–RAD51 signal disappearance was monitored over time. Image acquisition setup was performed as follows: 1.6 µW blue laser power, 0.1 ms px^−1^ dwell time, 100 nm pixel size, 1 s interframe wait time.

### Single-molecule DNA oligonucleotide capture assay

For all of the unwinding assays λ-ssDNA was held at constant force of 10 pN. Beads and DNA were kept in PBS during the experiment, while DNA was melted in 0.5× NTM buffer (25 mM Tris-HCl pH 7.5, 50 mM NaCl, 0.5 mM MgCl_2_) supplemented with 0.2 mg ml^−1^ BSA and 1 mM DTT in the presence of 5 nM RPA–eGFP. HELQ (5 nM), 5 nM RPA–eGFP and 2 nM 5′Cy3-(λ4)80 oligonucleotide (5′-Cy3-CCTGAACGACCAGGCGTCTTC GTTCATCTATCGGATCG CCACACTCACA ACAATGAGTG GCAGATATA GCCTGGTGGTTC-3′) or 2 nM 5′Cy3-dT79 oligonucleotide (5′-Cy3-TTTTTTTTTTTTTTTTTTTTTTTTTTTTTTTTTTTTTTTTTTTTTTTTTTTTTTTTTTTTTTTTTTTTTTTTTTTTTT-3′) was flowed into the system in 1× HELQ buffer (25 mM Tris-HCl pH 8.0, 2 mM MgCl_2_, 50 mM NaCl) supplemented with 2 mM ATP, 0.2 mg ml^−1^ BSA and 1 mM DTT. To directly image fluorescent oligonucleotide capture, the following image acquisition setup was used: 0.75 µW green laser power, 0.1 ms px^−1^ dwell-time, 100 nm pixel size, 0.5 s interframe wait time.

### Image processing and data analysis

Real-time force, distance and fluorescence data were exported from Bluelake HDF5 files and analysed using custom scripts in the Pylake Python package. Force was downsampled to 3 Hz for plotting. The worm-like chain model for λ-dsDNA was used as a reference for force–extension curve comparison. Bead distance–time traces were processed in GraphPad Prism 7. First derivative and smoothing of the traces were performed to extract individual unwinding stroke rates. Unwinding stroke rate distribution was analysed in GraphPad Prism 7 by fitting a single or double Gaussian curve. Dwell times and binding frequencies were estimated in Fiji. Dwell-time frequency distribution was analysed in GraphPad Prism 7. Mann–Whitney *U*-tests were used to assess statistical significance of the data where appropriate.

For the position, velocity and mean square displacement (MSD) analysis (Extended Data Fig. 4i–k), we used a custom-made single-particle tracking algorithm in Python (https://github.com/singlemoleculegroup). The sub-pixel position of the fluorescent particle in each frame of the kymograph was calculated by fitting the signal intensity of a three-frame moving window with a 1D Gaussian function (linetime = 0.997 s, 100 nm px^−1^).

For the obtained trajectories, the MSD was calculated using the following equation:

$${\rm{MSD}}(n,N)=\mathop{\sum }\limits_{i=1}^{N-n}\frac{{({X}_{i+n}-{X}_{i})}^{2}}{N-n}=D\varDelta {t}^{\alpha }$$

where *N* is the total number of frames in the kymograph, *n* is the number of frames within a moving window (lag time) from which the square displacement was calculated (ranging from 1 to *N *− 1) and *X*_*i*_ is the molecule position along the DNA in time.

To evaluate whether a trajectory represents random walk or directed motion, the MSD dependency was fitted with a power law. A particle exhibits free or constrained diffusion with rate *D* when the MSD scales with an exponent *α* ≤ 1. When *α* > 1, the process is characterized as superdiffusive motion (for example, unidirectional walk).

To estimate the average velocity of the translocating molecule, the total route of the molecule (a sum of frame-to-frame displacements) was divided by the total trajectory time. Here, every trajectory was smoothed using the Savitzky–Golay filter (smoothing factor = 51) to eliminate tracking inaccuracies and the molecule’s thermal fluctuations.

To estimate the loop size formed by the HELQ–RPA–DNA complex, the contour length after each unfolding event in the force–distance curve was fitted by the worm-like chain model. The difference between contour lengths of the neighbouring events corresponds with the loop size.

### RPA stripping using single-molecule FRET

Flow chambers were prepared as described previously^1,2^. Quartz slides and coverslips were passivated with polyethylene glycol (5% biotinylated) and flow chambers were constructed using double-sided sticky tape and sealed with epoxy. 5′-biotin- and internal amino linker-modified DNA oligonucleotides were labelled with Cy3-NHS ester and HPLC purified as previously described^3^. DNA (6 pM) was immobilized through biotin–streptavidin interactions. All of the experiments were performed in the standard HELQ buffer with addition of the PCA/PCD oxygen scavenger system with 5 mM PCA, 100 nM PCD and saturating Trolox. The flow chambers were imaged on a home-built, prism-based total internal reflection microscope with a 532 nm excitation laser (~3.8 mW), and images were acquired on an EM-CCD camera (Andor) with a 30 ms exposure time. FRET efficiencies were calculated from integrated donor (*I*_D_) and acceptor (*I*_A_) intensities as FRET =  *I*_A_/(*I*_D_ + *I*_A_) (refs. ^1,3^). The images and data were analysed using custom IDL, MATLAB and R scripts, which are available on request. FRET efficiency histograms were constructed by averaging the first 10 frames of each trajectory, with bins of 0.1. The dwell times of the free (high FRET) and bound (low FRET) states were measured, and dwell-time histograms were plotted. These were fit with single exponential fits to obtain average dwell times.

### Cell culture

The U2OS human osteosarcoma cell line was grown in DMEM supplemented with 10% bovine growth serum, 2 mM l-glutamine, 100 µg ml^−1^ streptomycin and 100 U ml^−1^ penicillin. U2OS-EJDR cells were cultured in DMEM supplemented with 10% tetracycline-free fetal bovine serum, 2 mM l-glutamine, 100 µg ml^−1^ streptomycin and 100 U ml^−1^ penicillin. U2OS-DR cells contain a stably integrated DR-GFP reporter to measure DSB repair by HR as previously described^35^. U2OS-SA cells contain a stably integrated SA-GFP reporter to measure DSB repair by SSA as previously described^36^. U2OS-DR cells containing a stably integrated EJ-RFP reporter to measure DSB repair by mutagenic end-joining constitute the U2OS-EJDR cell line as previously described^37^. U2OS-RFP-SCR cells contain a stably integrated RFP-SCR reporter for quantifying STGC and LTGC in HR as previously described^38^.

U2OS-DR *HELQ*^*−/−*^ and U2OS-SA *HELQ*^*−/−*^ cells were generated using the CRISPR–Cas9 system. Knockouts were verified using Sanger sequencing and immunoprecipitation/western blot.

### siRNA

The following siRNA oligonucleotides were used to transiently deplete *HELQ*: HELQ 1 (Qiagen FlexiTube siRNA, SI00435449); HELQ 2 (Horizon siGENOME SMARTpool, M-015379-01-0005); HELQ 3 (HelQ_M^23^; CAAAGGAAGATTTCCTCCAACTAAA).

*RAD52* was depleted using the On-Target plus SMART pool siRNA L-011760-00-0005 (Horizon). *BRCA2* was depleted using the On-Target plus SMART pool siRNA L-003462-00-0005 (Horizon). The On-Target plus non-targeting siRNA pool was used for non-targeting controls (D-001810-10-05, Horizon).

### DSB repair assays

Cells (0.25 × 10^6^) were reverse-transfected with 30 pmol siRNA using Lipofectamine RNAiMAX Reagent (Invitrogen) according to the manufacturer’s instructions. After 48 h, cells were transfected with 2 μg of pCMV-ISceI-3×NLS or pCMV 3×NLS empty vector and 30 pmol siRNA using Lipofectamine 2000 (Invitrogen). Cells were collected for analysis by flow cytometry at 72 h using the LSR Fortessa instrument (BD Biosciences). For each experiment, the percentage of GFP- or RFP-positive cells in the empty vector control was subtracted from the I-SceI-transfected cells. Data from each reporter assay represent the mean ± s.e.m. of at least three independent experiments, and statistical analysis was performed using two-tailed paired *t*-tests. The Cas9-mediated DSB repair assay was used to introduce a Cas9-mediated site-specific DSB and break repair outcomes were detected using next-generation sequencing as previously described^39^. In brief, cells were transfected with AAVS1 T2 CRISPR in pX330 (Addgene plasmid, 72833) using Lipofectamine 3000 (Invitrogen) and collected for genomic DNA extraction after 48 h. To measure SSTR, 1 µl of a 10 µM 121 bp donor oligonucleotide with three substitutions (purchased from IDT) was co-transfected with the CRISPR plasmid. A 201 bp PCR amplicon covering the expected Cas9 break site was sent for next-generation sequencing and reads were analysed for insertions, deletions and substitutions. NHEJ is defined as 1–5 bp deletion, MMEJ as >5 bp deletion with at least 2 bp microhomology, and SSTR as the introduction of three 1 bp substitutions. Data represent the mean ± s.e.m. of at least four independent experiments, and statistical analysis was performed using two-tailed paired *t*-tests.

### Immunoprecipitation and western blot

Cells were lysed in RIPA buffer (Teknova, R3792) with HALT protease inhibitor cocktail (Thermo Scientific Scientific). As HELQ is expressed at low levels in human cell lines and the commercially available antibodies tested did not dependably detect endogenous HELQ by western blot, we validated siRNA-mediated *HELQ* knockdown using HELQ immunoprecipitation. Cells (10^6^) were transfected with 2 µg siRNA by electroporation using the Amaxa nucleofector system, and plated into 150 mm dishes. After 72 h, cells were collected and whole-cell extracts were used for immunoprecipitation. Protein (2 mg) was incubated with 1 µg anti-HEL308 antibodies (Novus Biologicals, NBP1-91842) at 4 °C overnight with rotation. After washing 0.25 mg Pierce Protein A/G Magnetic Beads (Thermo Fisher Scientific, 88802), the antigen sample–antibody mix was added to the beads and incubated for 1 h at room temperature with rotation. The beads were washed four times and eluted in SDS–PAGE reducing sample buffer (Invitrogen) for 10 min at 96 °C. The samples were loaded onto 4–12% Bis-Tris precast gels (Invitrogen) for SDS–gel electrophoresis and transferred onto Immobilon-P PVDF membrane (Millipore). Membranes were blocked for 1 h at room temperature with Pierce clear milk blocking buffer. For western blot analysis of RAD52 and BRCA2, 50 μg of protein was loaded onto 10% Bis-Tris or 3–8% Tris-acetate precast gels (Invitrogen) for SDS–gel electrophoresis. Proteins were transferred onto a PVDF (polyvinylidene difluoride) membrane (BioRad) for BRCA2 detection. The iBlot Gel Transfer System (Invitrogen) was used to perform dry blotting of proteins onto nitrocellulose membranes for RAD52 detection. Membranes were blocked for 1 h at room temperature with Pierce clear milk blocking buffer. Antibodies for the western blot analysis were as follows: anti-HEL308 (Santa Cruz Biotechnology, sc-81095), anti-RAD52 (Santa Cruz Biotechnology, sc-365341), anti-BRCA2 Ab-1 (Millipore Sigma-Aldrich, OP95), anti-SMC1 (Bethyl laboratories, A300-055A).

### Gene expression

Cells were collected 72 h after siRNA transfection. Cell lysis, reverse transcription and quantitative PCR were performed using the TaqMan Gene Expression Cells-to-CT kit from Invitrogen according to the manufacturer’s instructions. TaqMan Gene Expression Assays for HELQ (Hs01127906_m1) and ACTB endogenous control (Hs99999903_m1) were run in triplicate on the QuantStudio 6 Pro real-time PCR instrument (Applied Biosystems). Relative fold gene expression was calculated using the ∆∆*C*_t_ method. Data represent the mean ± s.e.m. of at least three independent experiments, and statistical analysis was performed using two-tailed paired *t*-tests.

### Immunofluorescence

WT U2OS-DR and *HELQ*^−/−^ U2OS-DR cells were seeded onto four-chamber tissue culture slides (Millipore) and treated the next day with 4 µM camptothecin or 10 Gy irradiation. For siRNA knockdown, cells were transfected with non-targeting or *HELQ* siRNA, incubated overnight and seeded onto chamber slides the next day. Cells were treated with the designated damaging agent 48 h after siRNA transfection. At the designated time points, cells were fixed, blocked and permeabilized. Cells were stained with the following antibodies: anti-phosphorylated-histone H2A.X (Ser139) (05-636, Millipore Sigma-Aldrich), anti-RAD51 (PC130 Millipore Sigma-Aldrich), anti-RPA32 (2208, Cell Signaling Technology), anti-phosphorylated-RPA32 (S4/S8) (ab87277, Abcam). Secondary antibodies were as follows: AlexaFluor 488-labelled goat anti-rabbit IgG, AlexaFluor 568-labelled donkey anti-mouse IgG, AlexaFluor 568-labelled goat anti-rat (Invitrogen). Images were obtained using a Zeiss LSM 880 confocal laser scanning microscope and analysed using ImageJ. At least 100 nuclei were counted per experiment and nuclei with >5 foci were scored as positive. Data represent the mean ± s.e.m. of at least three independent experiments, and statistical analysis was performed using two-tailed paired *t*-tests.

### Software

Chemidoc MP Image Lab Touch software (Bio-Rad, v.2.2.0.08) was used for gel imaging. Quenching-based kinetic unwinding assay data were collected using Clariostar BML Labtech (v.5.20 R5). Bluelake software from LUMICKS was used for data collection of SMI (LUMICKS). Similarly, for smFRET-based assays, NimOS software from ONI was used. BD FACSDiva software was used with the BD Biosciences LSR Fortessa Analyzer for flow cytometry data acquisition. Zen 2.3 SP1 FP3 (black) v.14.0.18.201 was used for confocal microscopy image acquisition.

The quantification of gel-based data was carried using ImageJ (NIH v.1.52k). Mars Data analysis software (BML Labtech v.3.10 R6) was used for quenching-based kinetic unwinding assays. To analyse the SMI by optical tweezer, Pylake software from Lumicks was used. Furthermore, custom scripts were used for analysing some of unwinding/translocation assays carried out by SMI (https://github.com/singlemoleculegroup). smFRET analysis was performed using iSMS software (open source)^39^. For microscopy data analysis, Image J (NIH, v.1.53e) was used. QuantStudio Design and Analysis Software v.2 was used with the QuantStudio 6 Pro real-time PCR instrument for relative gene expression analysis. Flow cytometry data were analysed using BD FlowJo (v.10.6.2). FlowJo was used to gate single cells and then select for GFP^+^ and/or RFP cells depending on the assay as shown. The background signal from the untransfected control was subtracted from each experiment. Representative plots from FlowJo showing the gating strategy are provided in Supplementary Fig. 2. Cas9 DSB repair assay sequencing data were analysed as in Hussain et al.^40^, using PEAR software for read stitching, BLOSUM62 for alignment and code for microhomology/deletion analysis available on GitHub (https://github.com/cjsifuen/delmh). Statistical analysis was performed using GraphPad Prism (v.8.2.1 and v.8.4.2). All schematics except for those of the quenching-based kinetic unwinding assays were generated using Adobe illustrator v.2.3. For the schematics of quenching-based kinetic unwinding assays, Biorender.com was used.

### Reporting summary

Further information on research design is available in the Nature Research Reporting Summary linked to this paper.

## Online content

Any methods, additional references, Nature Research reporting summaries, source data, extended data, supplementary information, acknowledgements, peer review information; details of author contributions and competing interests; and statements of data and code availability are available at 10.1038/s41586-021-04261-0.

## Supplementary information

Supplementary Information Supplementary Figs. 1 and 2.

Reporting Summary

## Data Availability

The datasets generated during and/or analysed during the current study are included alongside the Article. Raw datasets for experiments performed on the C-TRAP are not included and are available from S.J.B. on reasonable request. Gel source data are provided in the Supplementary Information. All data are archived at the Francis Crick Institute or Sloan Kettering Institute.

## Code availability

The custom script made for determining the DNA unwinding/translocation by SMI is available at GitHub (https://github.com/singlemoleculegroup).
