# Supplementary material for: HELQ is a dual-function DSB repair enzyme modulated by RPA and RAD51
Source: Nature. 2021 Dec 22;601(7892):268–73. doi: 10.1038/s41586-021-04261-0 (PMC8755542; doi:10.1038/s41586-021-04261-0)
Supplement: Supplementary file 1 — Supplementary Figs. 1 and 2. [file 41586_2021_4261_MOESM1_ESM.pdf]

nature portfolio

https://doi.org/10.1038/s41586-021-04261-0

Supplementary information

HELQ is a dual-function DSB repair enzyme modulated by RPA and RAD51

In the format provided by the authors and unedited

Supplementary Information

HELQ is a dual function DSB repair enzyme modulated by RPA and RAD51

Roopesh Anand[1,5], Erika Buechelmaier[2,5], Ondrej Belan[1], Matthew Newton[1], Aleksandra Vancevska[1], Artur Kaczmarczyk[3,4], Tohru Takaki[1], David S. Rueda[3,4, 6], Simon N. Powell[2,6], Simon J. Boulton[1,6]

Contents:

SI Figure 1
p1: Legend
p2: source data for Fig 1a, c and f
p3: source data for Fig 2a, c, e and g
p4: source data for Fig3i
p5: source data for EDFig 1a, b, c, d, f, g, h, i, j and l
p6: source data for EDFig 2a, b, c, d, e, f, h, i, j and k
p7: source data for EDFig 2m and o
p8: source data for EDFig 3a, b, d, f and h
p9: source data for EDFig 4h
p10: source data for EDFig 5a, c, e, g, h, j and l
p11: source data for EDFig 6a, b, and q
p12: source data for EDFig 7a and b
p13: source data for EDFig 8a and d
p14: source data for EDFig 9a, b and f

SI Figure 2
p15: legend
p16: FACs gating strategy for U2OS-DR
p17: FACs gating strategy for U2OS-SA and U2OS-SCR
p18: FACs gating strategy for EJ-DR

SI Figure 1
Legend:
Source data for Figures 1-4 and EDFigure 1-8.
This shows images of the uncropped gels and the cropped section used for the indicated main or ED figures. Molecular weight markers are not included as these are DNA gels.

Source data for EDFigure 9.
This shows scanned in uncropped western blots and the cropped section used for the indicated ED figures. The antibody used/protein detected is shown and the molecular weight markers are included.

Fig. 1a

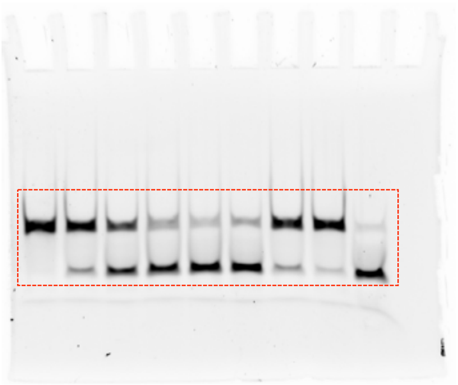

Fig. 1c

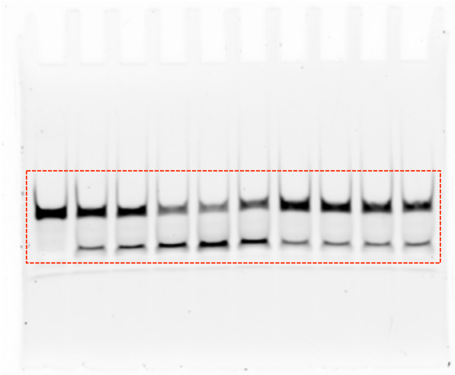

Fig. 1f

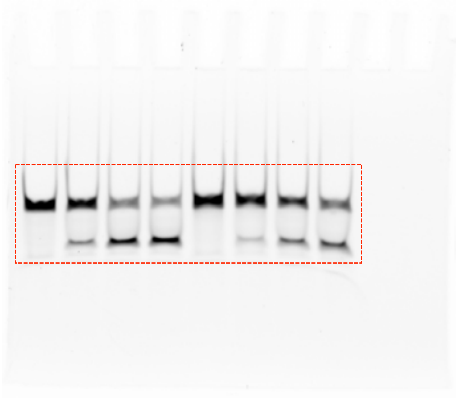

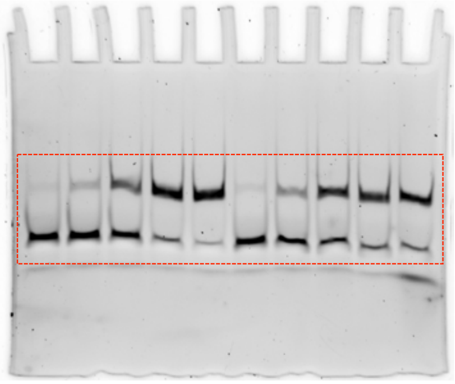

Fig. 2a

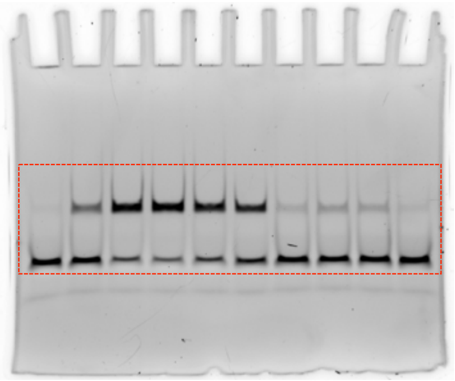

Fig. 2c

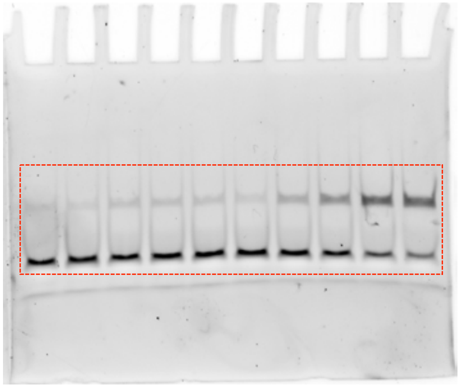

Fig. 2e

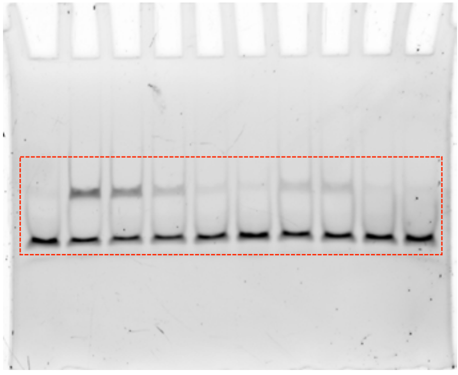

Fig. 2g

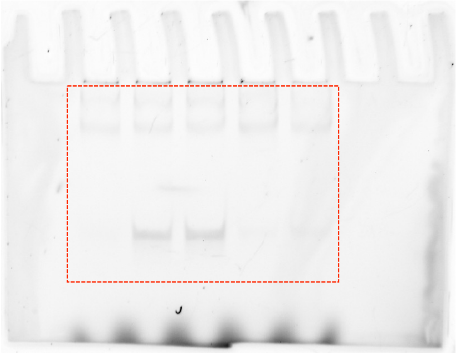

Fig. 3 i

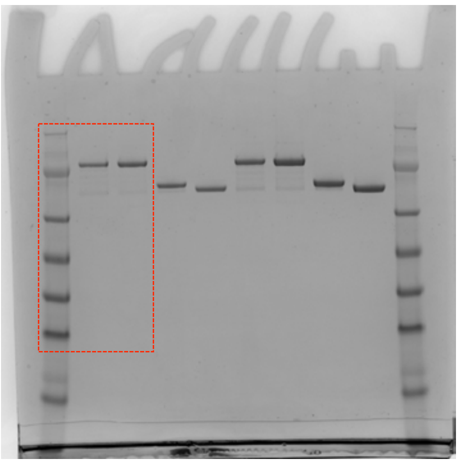
ED Fig. 1a

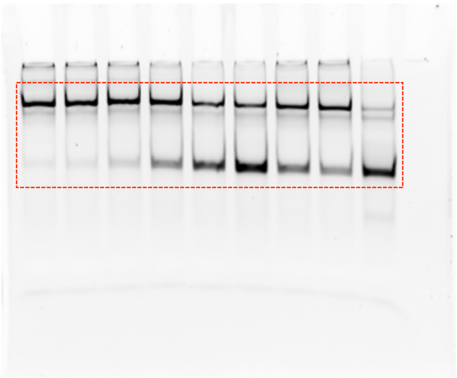
ED Fig. 1b

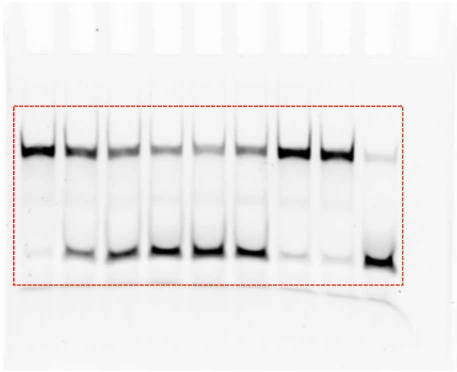
ED Fig. 1c

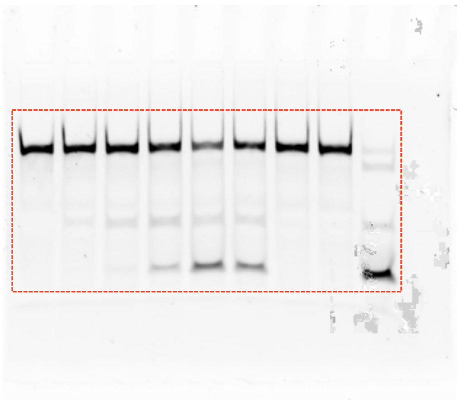
ED Fig. 1d

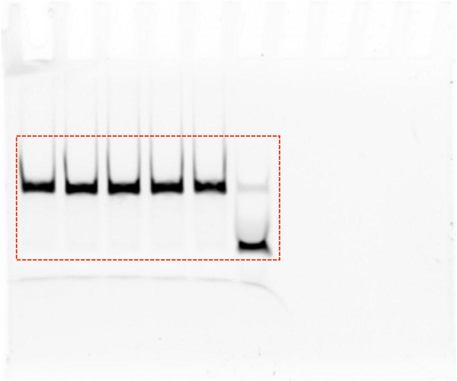
ED Fig. 1f

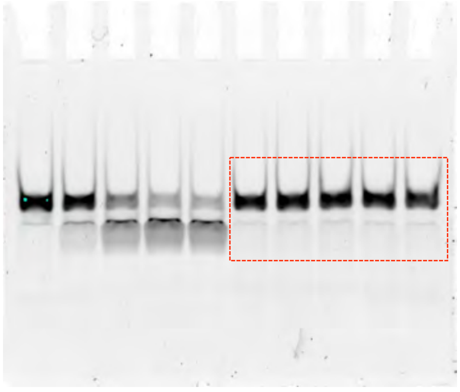
ED Fig. 1g

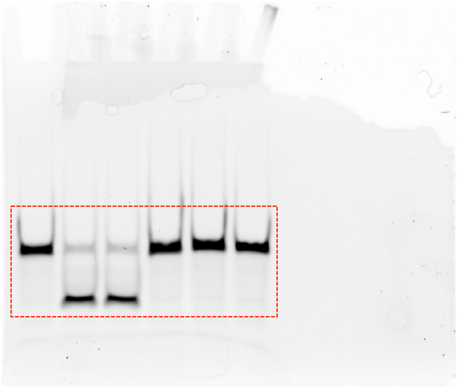
ED Fig. 1h

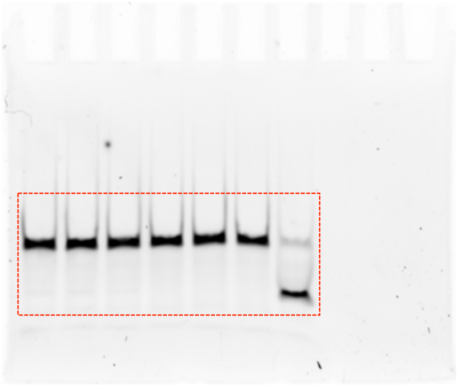
ED Fig. 1i

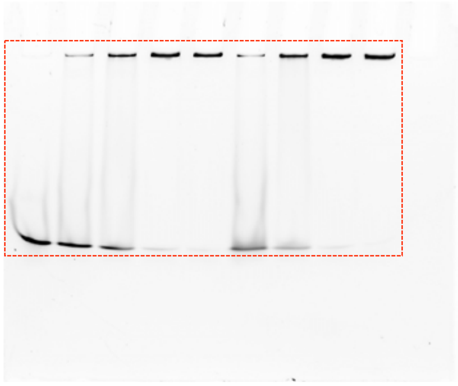
ED Fig. 1j

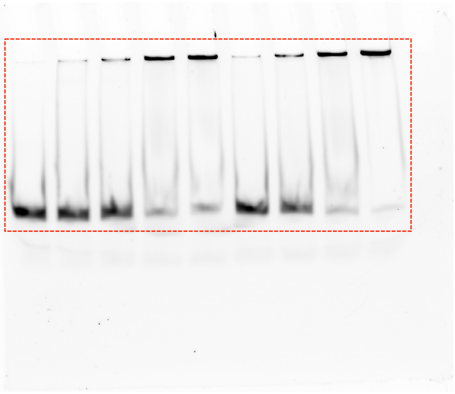
ED Fig. 1l

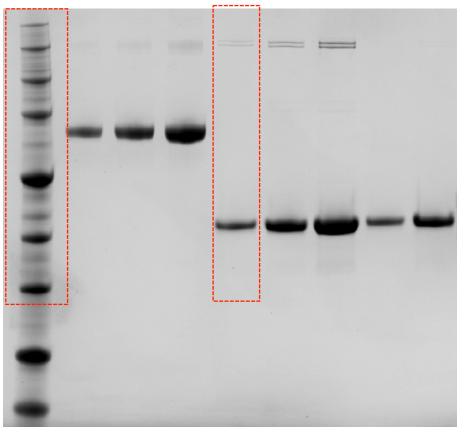
ED Fig. 2a

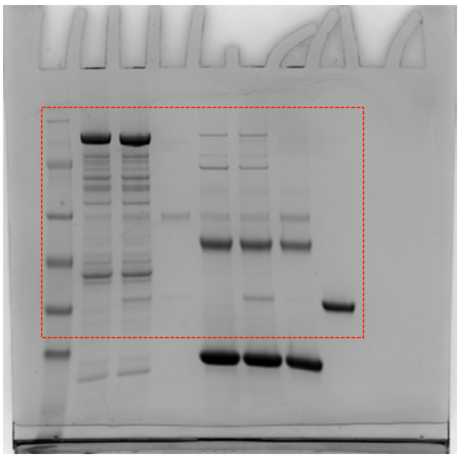
ED Fig. 2b

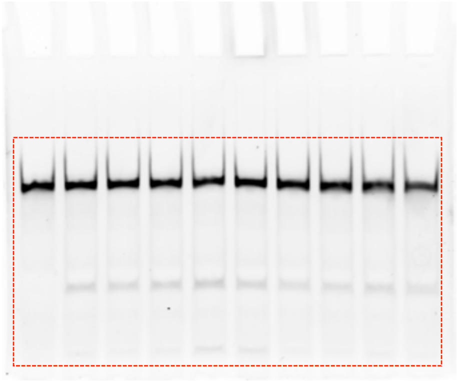
ED Fig. 2c

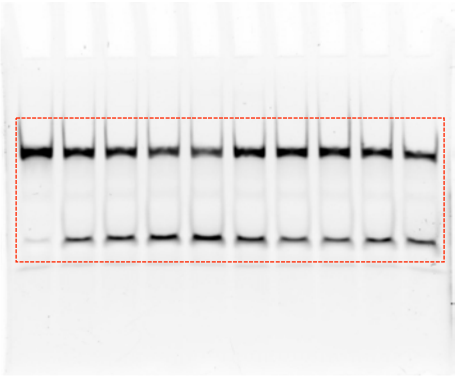
ED Fig. 2d

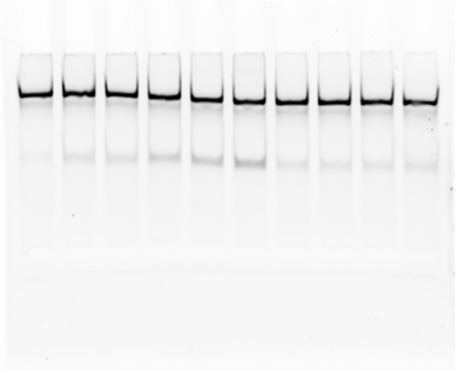
ED Fig. 2e

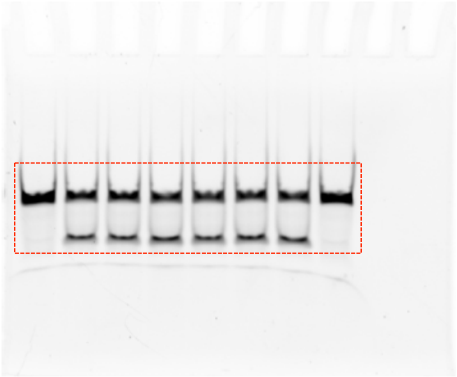
ED Fig. 2f

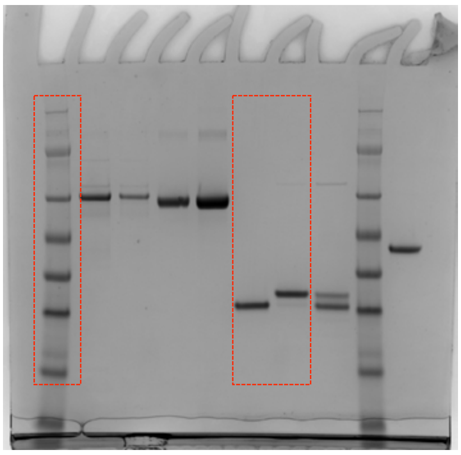
ED Fig. 2h

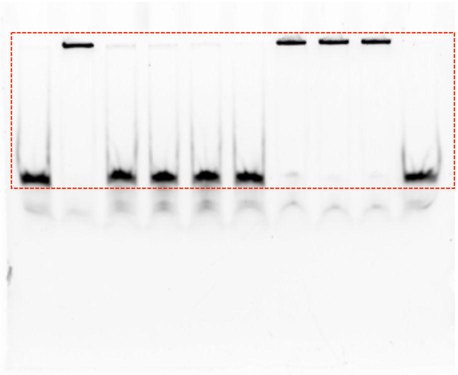
ED Fig. 2i

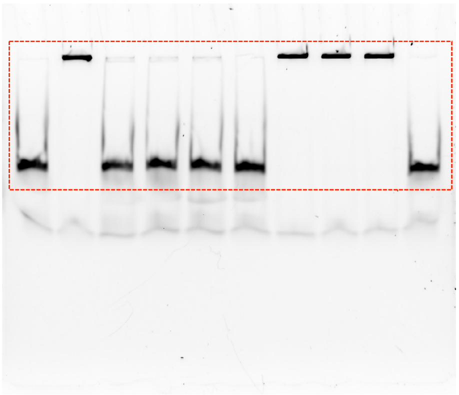
ED Fig. 2j

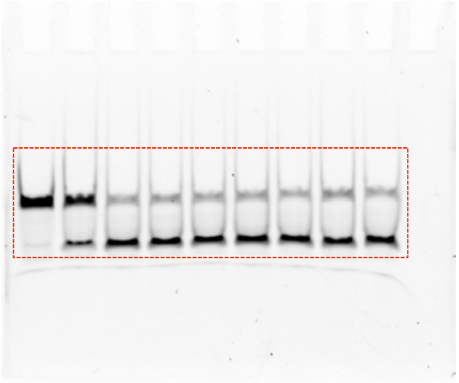
ED Fig. 2k

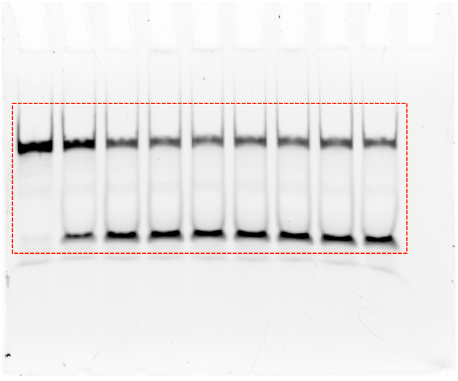

ED Fig. 2m

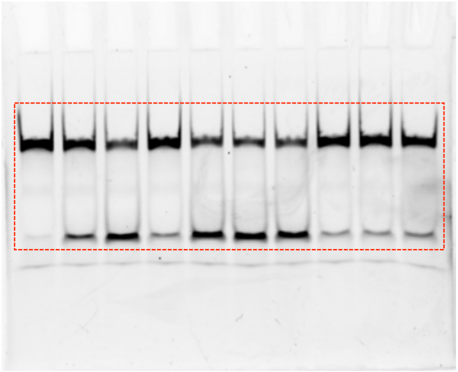

ED Fig. 2o

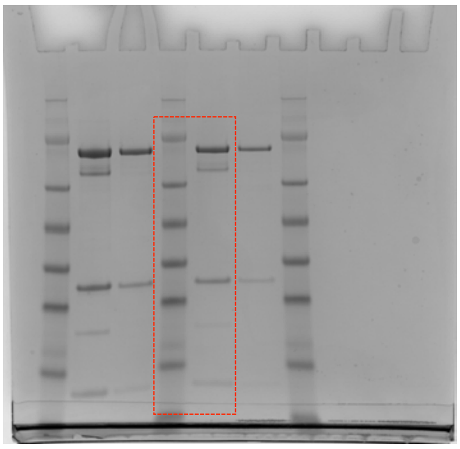

ED Fig. 3a

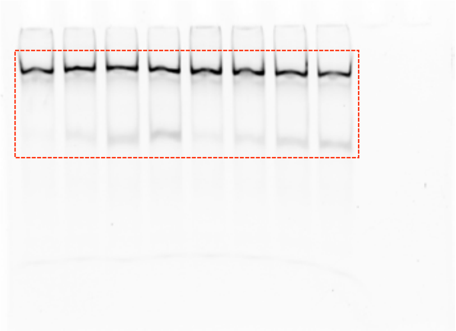

ED Fig. 3b

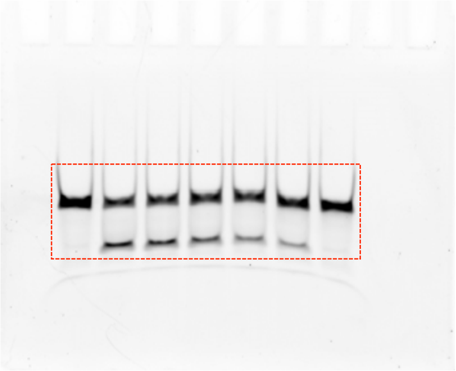

ED Fig. 3d

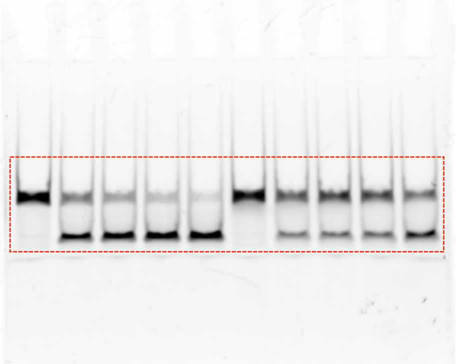

ED Fig. 3f

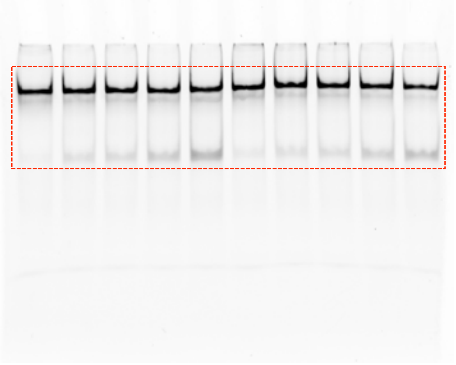

ED Fig. 3h

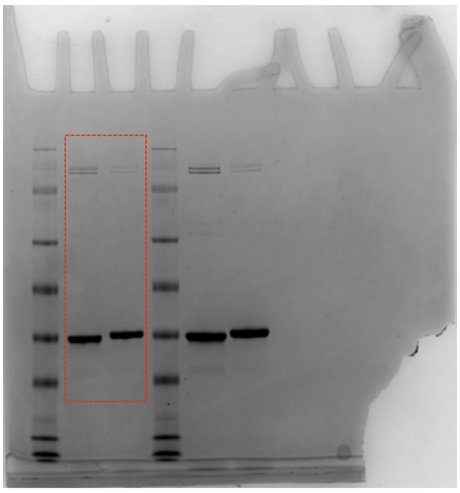

CBB

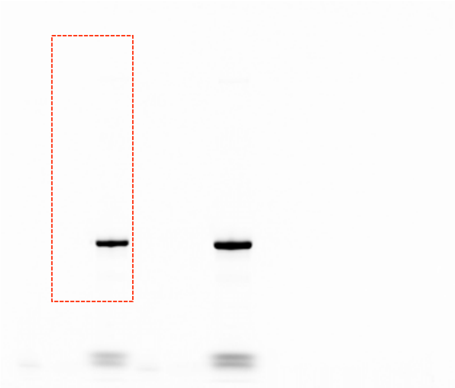

A488

ED Fig. 4h

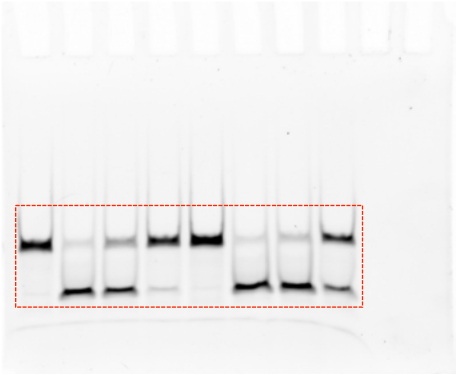
ED Fig. 5a

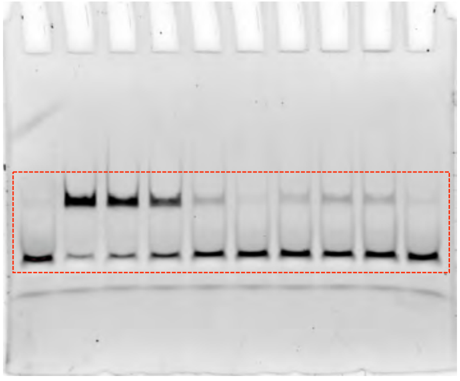
ED Fig. 5j

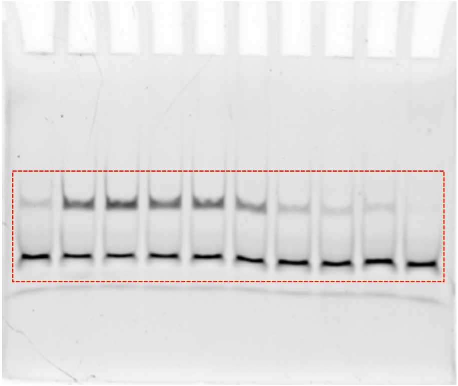
ED Fig. 5c

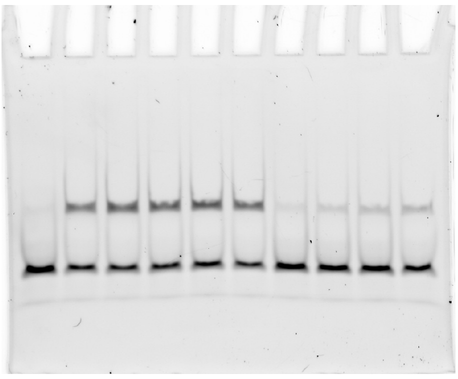
ED Fig. 5l

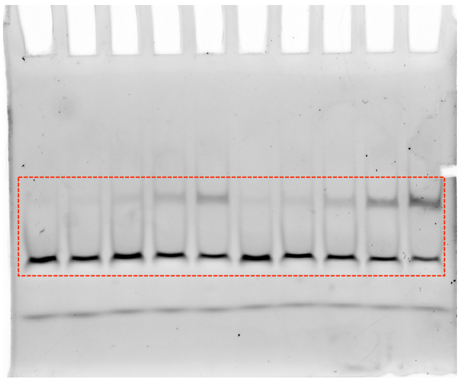
ED Fig. 5e

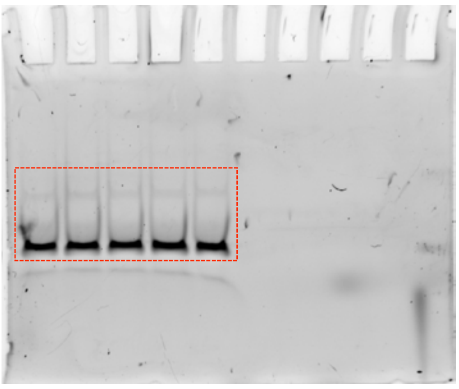
ED Fig. 5g

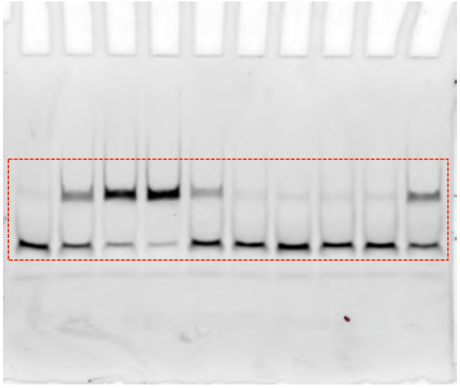
ED Fig. 5h

ED Fig. 6b

ED Fig. 6a

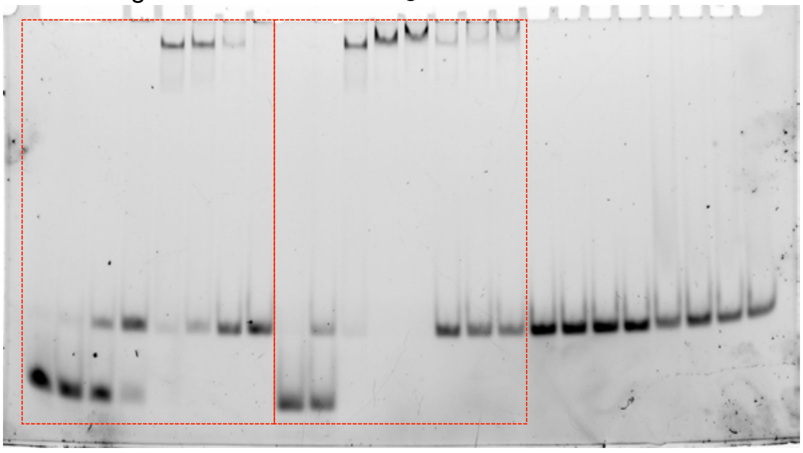

ED Fig. 6a,b (Top)

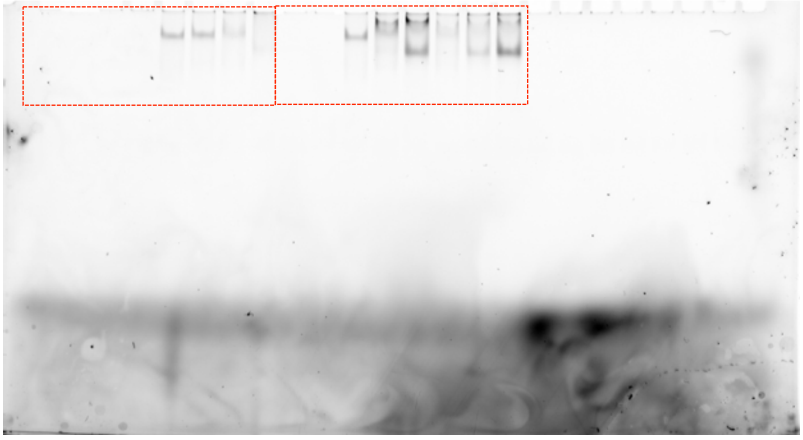

ED Fig. 6a,b (Bottom)

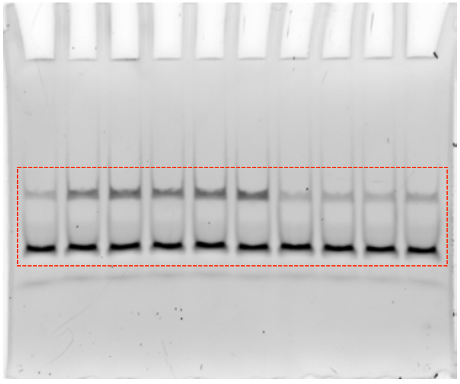

ED Fig. 6q

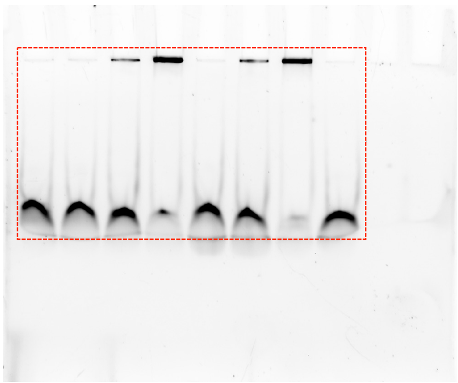

ED Fig. 7a

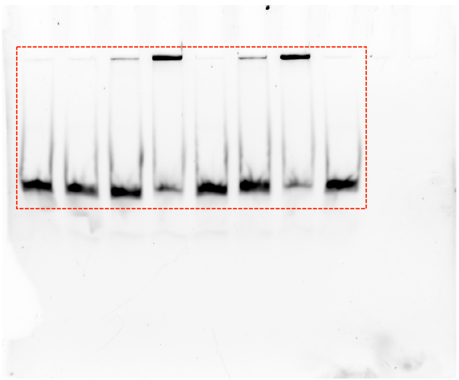

ED Fig. 7b

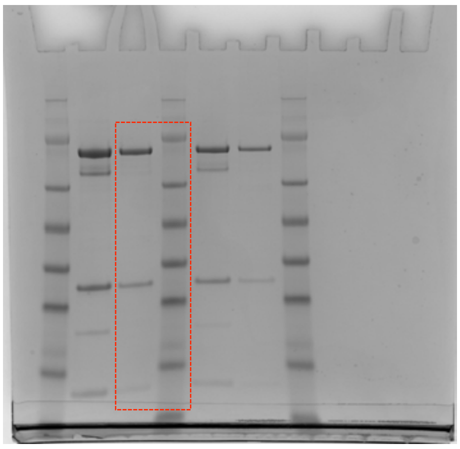

ED Fig. 8a (same protein ladder lane used as shown in ED Fig. 3a)

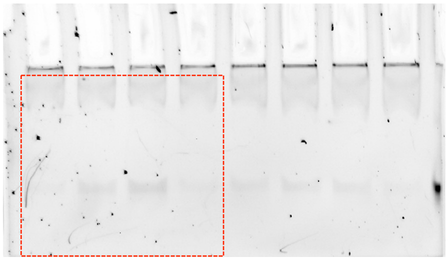

ED Fig. 8d

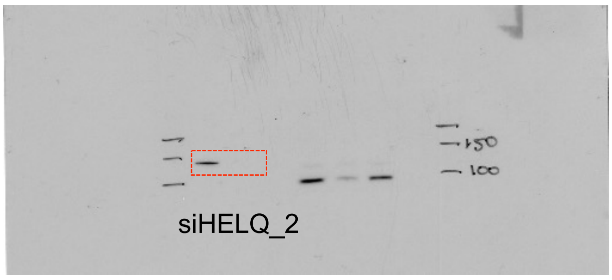

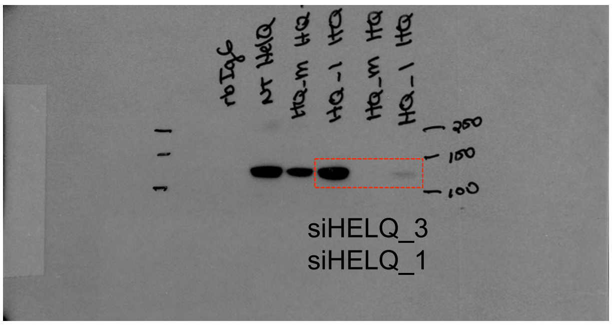

ED Fig. 9a

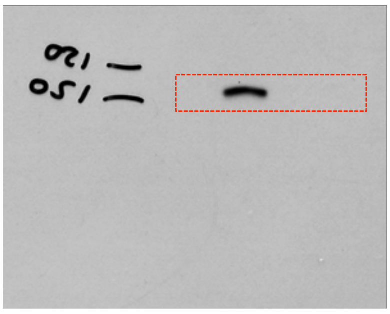

ED Fig. 9b

Short exposure

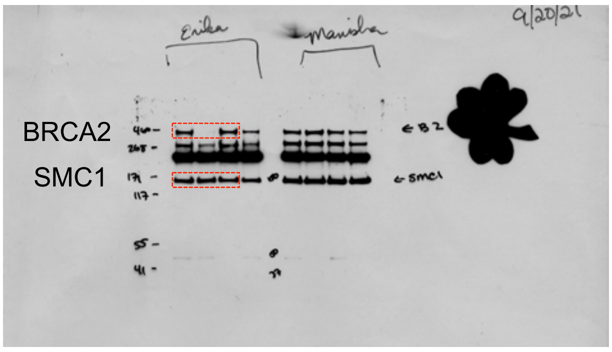

Long exposure

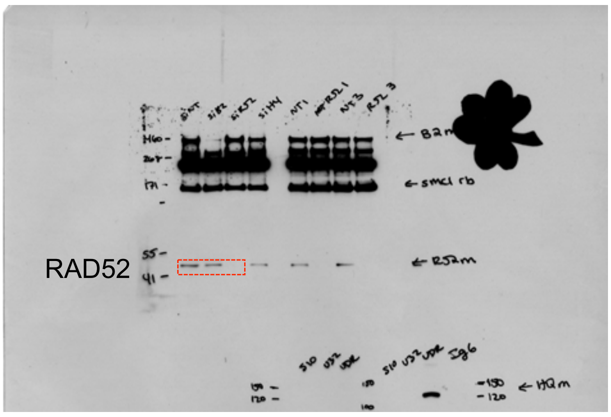

ED Fig. 9f

SI Figure 2
Legend:

Shown are representative plots from FlowJo with the FACs gating strategy for U2OS-DR, U2OS-SA, U2OS-SCR and EJ-DR

U2OS-DR

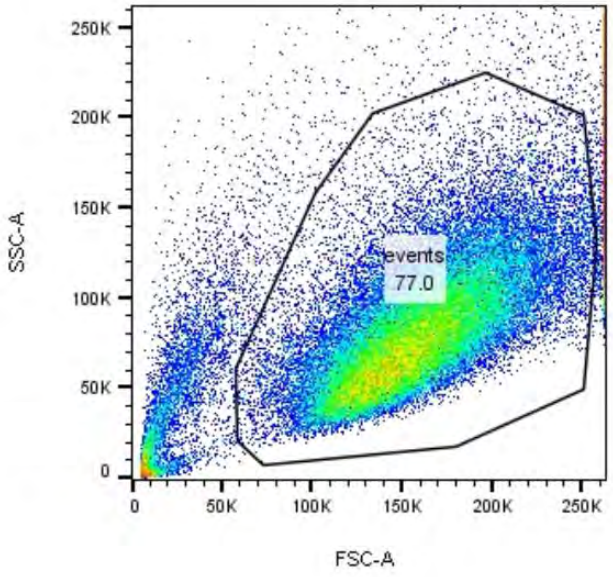

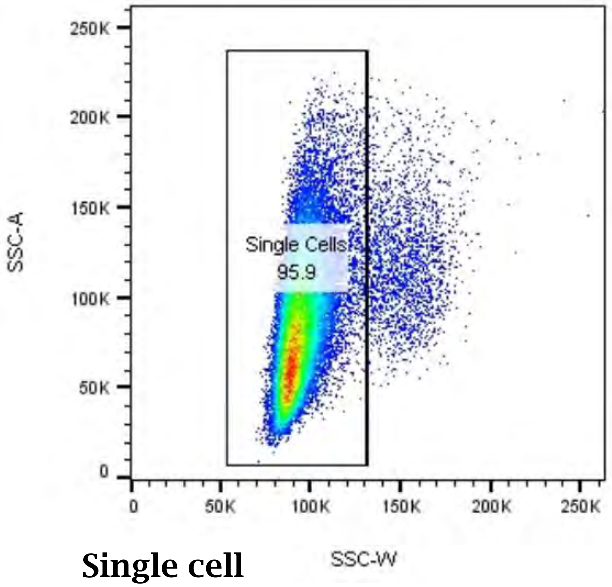

Single cell gating

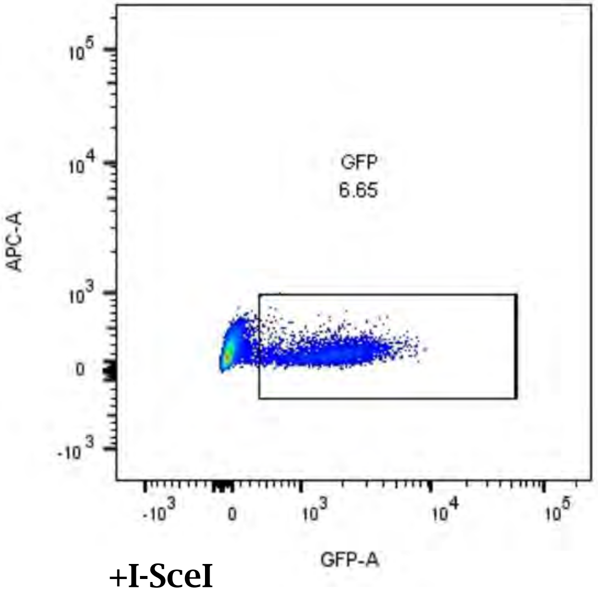

+I-SceI

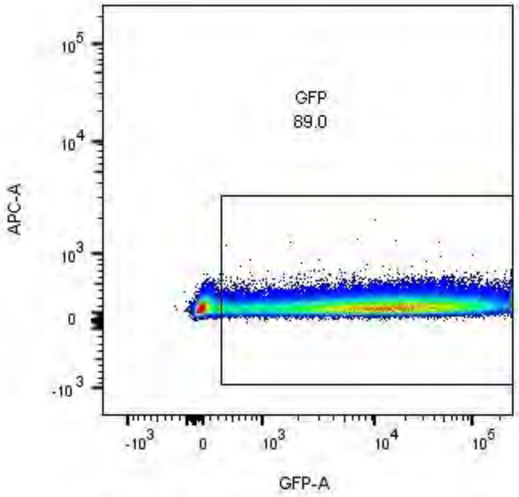

GFP positive control

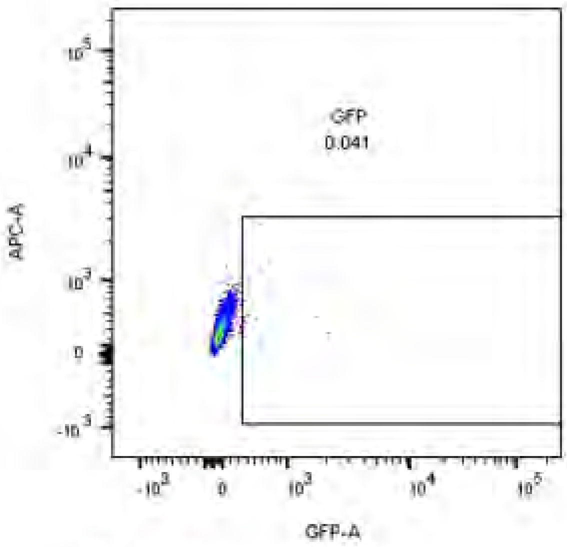

-I-SceI

U2OS-SA

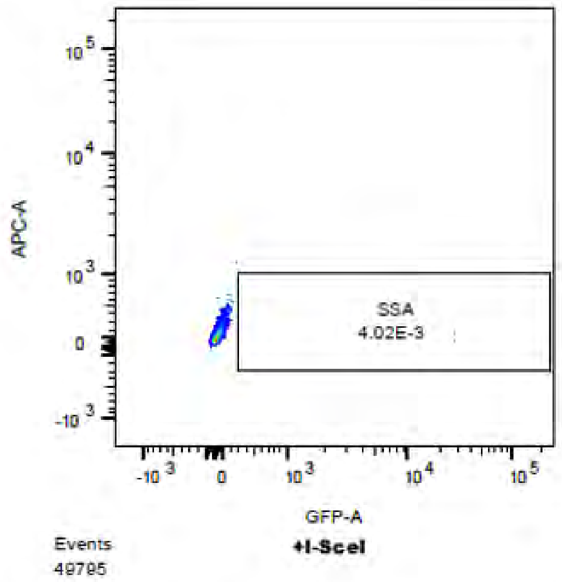

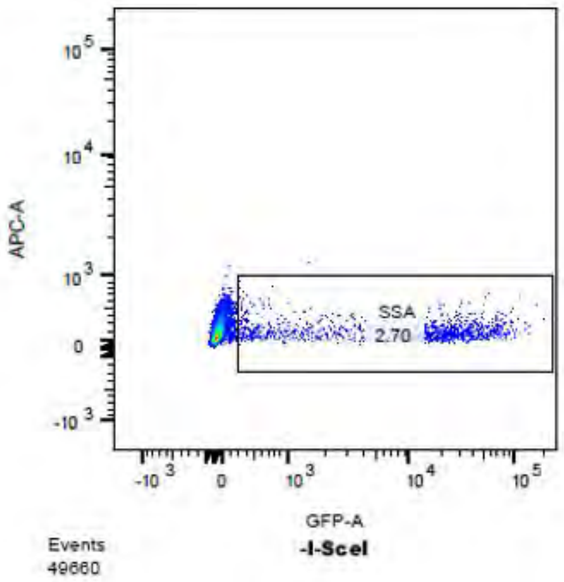

U2OS-SCR

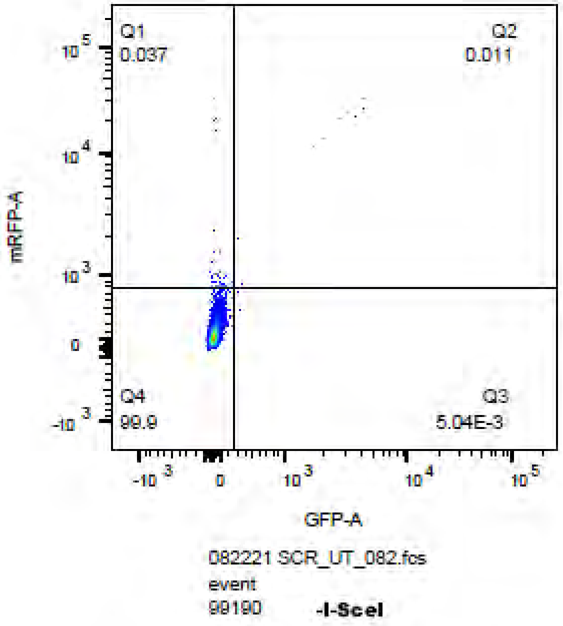

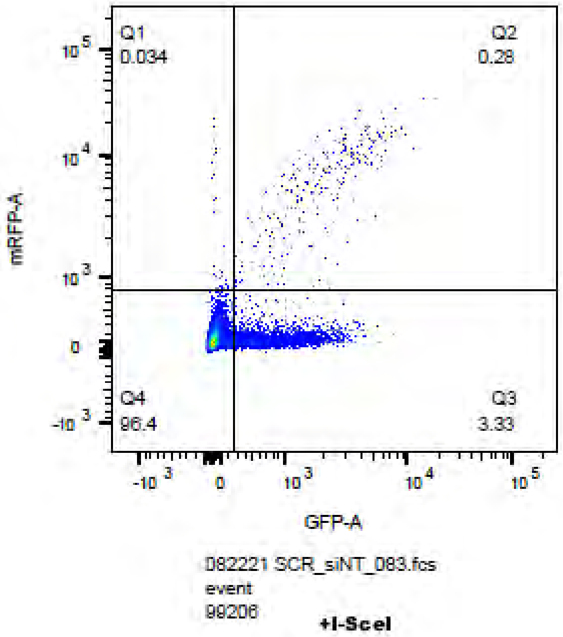

EJ-DR

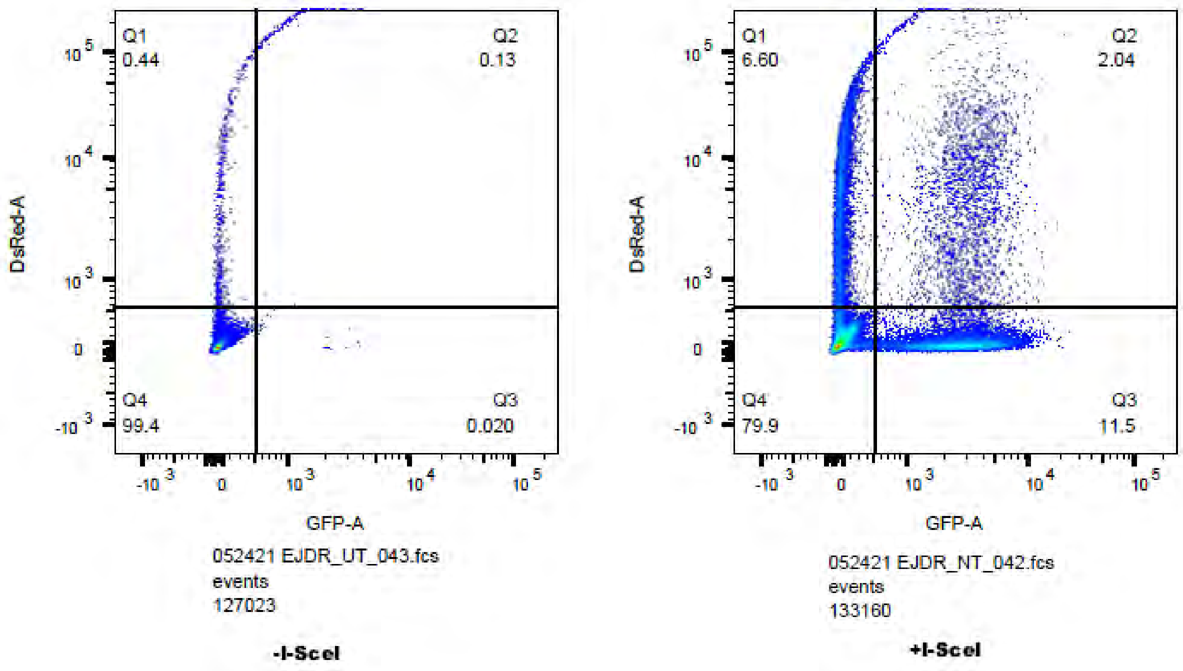
Q1
0.44
Q2
0.13
Q4
99.4
Q3
0.020
DsRed-A
GFP-A
052421 EJDR_UT_043.fcs
events
127023
-I-SceI
Q1
6.60
Q2
2.04
Q4
79.9
Q3
11.5
DsRed-A
GFP-A
052421 EJDR_NT_042.fcs
events
133160
+I-SceI
