# Supplementary material for: HELQ is a dual-function DSB repair enzyme modulated by RPA and RAD51
Source: Nature. 2021 Dec 22;601(7892):268–73. doi: 10.1038/s41586-021-04261-0 (PMC8755542; doi:10.1038/s41586-021-04261-0)
Supplement: Supplementary file 2 — Reporting Summary [file 41586_2021_4261_MOESM2_ESM.pdf]

nature portfolio

Corresponding author(s): *Double-blind peer review submissions: write DBPR and your manuscript number here instead of author names.*

Last updated by author(s): *YYYY-MM-DD*

Reporting Summary

Nature Portfolio wishes to improve the reproducibility of the work that we publish. This form provides structure for consistency and transparency in reporting. For further information on Nature Portfolio policies, see our Editorial Policies and the Editorial Policy Checklist.

Statistics

For all statistical analyses, confirm that the following items are present in the figure legend, table legend, main text, or Methods section.

n/a	Confirmed	
☐	☒	The exact sample size (n) for each experimental group/condition, given as a discrete number and unit of measurement
☐	☒	A statement on whether measurements were taken from distinct samples or whether the same sample was measured repeatedly
☐	☒	The statistical test(s) used AND whether they are one- or two-sided<br>*Only common tests should be described solely by name; describe more complex techniques in the Methods section.*
☒	☐	A description of all covariates tested
☒	☐	A description of any assumptions or corrections, such as tests of normality and adjustment for multiple comparisons
☐	☒	A full description of the statistical parameters including central tendency (e.g. means) or other basic estimates (e.g. regression coefficient) AND variation (e.g. standard deviation) or associated estimates of uncertainty (e.g. confidence intervals)
☐	☒	For null hypothesis testing, the test statistic (e.g. F, t, r) with confidence intervals, effect sizes, degrees of freedom and P value noted<br>*Give P values as exact values whenever suitable.*
☒	☐	For Bayesian analysis, information on the choice of priors and Markov chain Monte Carlo settings
☒	☐	For hierarchical and complex designs, identification of the appropriate level for tests and full reporting of outcomes
☒	☐	Estimates of effect sizes (e.g. Cohen's d, Pearson's r), indicating how they were calculated

Our web collection on statistics for biologists contains articles on many of the points above.

Software and code

Policy information about availability of computer code

Data collection	We used commercial software available with the respective instruments for data collection. These include Chemidoc MP Image Lab Touch Software, Biorad (version 2.2.0.08) for gel imaging, Clariostar BML Labtech (version 5.20 R5) for Quenching-based kinetic assay for DNA unwinding, Bluelake software for single-molecule imaging (LUMICKS), NimOS software (ONI) Version 1.16.4.13788 for smFRET, BD FACSDiva software (v8.0.1) was used with BD Biosciences LSR Fortessa analyzer for flow cytometry data acquisition; Zen 2.3 SP1 FP3 (black) v14.0.18.201 was used for confocal microscopy image acquisition.
Data analysis	We used combination of custom made, commercial or publicly available software used for data analysis. These include ImageJ (NIH Version 1.52k) for quantifying gel based assays, Mars Data analysis software (BML Labtech version 3.10 R6), Pylake software (Lumicks) for single-molecule imaging by optical tweezer, Custom script made for some of DNA unwinding/translocation SMI assay (https://github.com/singlemoleculegroup), iSMS software (open source) for smFRET analysis (Preus et al. iSMS: single-molecule FRET microscopy software. Nat Methods 12, 593–594 (2015)), Image J (NIH, Version 1.53e) for the analysis of microscopy data; graphs and numerical data (including statistics/error bars) was analyzed and plotted by Prism (GraphPad, Version 8.2.1 and 8.4.2); flow cytometry data was analyzed using FlowJo v10.6.2; QuantStudio Design and Analysis Software v2 was used with QuantStudio 6 Pro real-time PCR instrument for relative gene expression analysis; Cas9 DSB repair assay sequencing data was analyzed as in Hussain et al, Nucleic Acids Research 2021, using PEAR software for read stitching, BLOSUM62 for alignment, and code for microhomology/deletion analysis available on Github https://github.com/cjsifuen/delmh.

For manuscripts utilizing custom algorithms or software that are central to the research but not yet described in published literature, software must be made available to editors and reviewers. We strongly encourage code deposition in a community repository (e.g. GitHub). See the Nature Portfolio guidelines for submitting code & software for further information.

Data

Policy information about availability of data

All manuscripts must include a data availability statement. This statement should provide the following information, where applicable:

- Accession codes, unique identifiers, or web links for publicly available datasets
- A description of any restrictions on data availability
- For clinical datasets or third party data, please ensure that the statement adheres to our policy

The datasets generated during and/or analysed during the current study are included alongside the Article or are available from S.J.B and S.P on reasonable request. For gel source data, see source data file. All data are archived at the Francis Crick Institute or Sloan Kettering Institute. The custom script made for determining the DNA unwinding/translocation by SMI is available at link "https://github.com/singlemoleculegroup".

Field-specific reporting

Please select the one below that is the best fit for your research. If you are not sure, read the appropriate sections before making your selection.

☒ Life sciences ☐ Behavioural & social sciences ☐ Ecological, evolutionary & environmental sciences

For a reference copy of the document with all sections, see nature.com/documents/nr-reporting-summary-flat.pdf

Life sciences study design

All studies must disclose on these points even when the disclosure is negative.

Sample size	The sample size was determined according to standard practices of field of biochemistry and Cell biology and what was pragmatic number to testing to carry out. Wherever quantification is provided, minimum 3 independent experiments were carried out to perform statistical analysis, As per field practices, for all experiments showing quantification bar/graphs, experiments were repeated at 3 (or more) times.
Data exclusions	In general, no data was excluded except where experiments failed due to failed control and technical problems.
Replication	To ensure the replication, each experiment was performed multiple times in exact condition on different days. For protein-protein interaction, interaction assay was repeated thrice with slightly different conditions and all showed the same result indicating the robustness of the result. Some gels were not included in the study due to technical failure. Any result, which did not replicate was not included in the study.
Randomization	Randomization is not relevant to the kind of experiments performed in this study because randomization of samples is not applicable to cell lines and in vitro studies. On the contrary, researcher needs to know every sample and assay to carry out these kinds of experiments.
Blinding	Blinding is not relevant to the kind of experiments performed in this study because of the same above reason. For example, a researcher exactly needs to know which protein he/she is working with to carry out relevant assays to understand their functions. The same is applicable to cell lines and thus cell biology data. Just like randomization, this is not at all applicable to these kinds of experiments.

Reporting for specific materials, systems and methods

We require information from authors about some types of materials, experimental systems and methods used in many studies. Here, indicate whether each material, system or method listed is relevant to your study. If you are not sure if a list item applies to your research, read the appropriate section before selecting a response.

Materials & experimental systems

n/a	Involved in the study	
☐	☒	Antibodies
☐	☒	Eukaryotic cell lines
☒	☐	Palaeontology and archaeology
☒	☐	Animals and other organisms
☒	☐	Human research participants
☒	☐	Clinical data
☒	☐	Dual use research of concern

Methods

n/a	Involved in the study	
☒	☐	ChIP-seq
☒	☐	Flow cytometry
☒	☐	MRI-based neuroimaging

Antibodies

Antibodies used	For Western blot, we used the following antibodies. anti-HEL308 (2406C1a, Santa Cruz Biotechnology, sc-81095, lot#J1217, 1:200 dilution), anti-RAD52 (F-7, Santa Cruz Biotechnology, sc-365341, lot#I2616, dilution 1:500), anti-BRCA2 Ab-1 (clone 2B, Millipore Sigma, OP95, lot#3011827, dilution 1:300), anti-SMC1 (Bethyl laboratories, A300-055A, lot#6, dilution1:1000). For immunoprecipitation of HELQ, we used Hel308 (Novus Biologicals NBP1-91842, lot#A91998). For IF studies, Cells were stained with

the following antibodies: anti-phospho-histone H2A.X (Ser139) (clone JBW301, 05-636, Millipore Sigma, lot#3292608, dilution 1:500), anti-RAD51 (Ab-1, PC130 Calbiochem Millipore Sigma, lot#3092494, dilution 1:500), anti-RPA32 (4E4, #2208, Cell Signaling Technology, lot#5, dilution 1:400), anti-phospho-RPA32 (S4/S8) (ab87277, Abcam, lot#GR3182765-31, dilution 1:200).AlexaFlour 488-labeled goat anti-rabbit IgG (Invitrogen, A11008, dilution 1:1000), AlexaFlour 568-labeled donkey anti-mouse IgG (Invitrogen, A10037, dilution 1:1000)
AlexaFlour 568-labeled goat anti-rat (Invitrogen, A11077, dilution 1:1000).

Validation

The specificity of anti-HEL308 (Santa Cruz Biotechnology, sc-81095) was validated in Liu, DN. et al. 2017. Oncol. Rep. 37: 1107-1113.It was also validated by manufacturer in human cells for WB (https://datasheets.scbt.com/sc-81095.pdf) . anti-RAD52 (Santa Cruz Biotechnology, sc-365341) specificity was determined previously in multiple studies including most recent research articles Kilgas et al. 2021. Cell Rep. 35: 109153 and Zhu et al. 2021. NAR Cancer. 3: zcab010. It was also validated by manufacturer in human cells for WB (https://datasheets.scbt.com/sc-365341.pdf). anti-BRCA2 (Millipore Sigma, OP95) has validated in numerous studies including the most recents studies Ghouil et al. Nat Commun 12, 4605 (2021).It was also validated by manufacturer in human cells for WB. anti-SMC1 was used only as loading control in western blot and has been validated by Bethyl Laboratories for WB (https://www.bethyl.com/product/A300-055A?referrer=search). anti-HEL308 (Novus Biologicals, NBP1-91842) specificity was determined the manufacturer where they tested the specificity by protein array containing target protein and other 383 non-specific proteins. anti-phospho-hostome H2A.X (Ser139) (Millipore Sigma, 05-636) was validated in Xe, X., et al. (2015) Nat. Cell Biol. 20 (3); 320-331. It was also validated by manufacturer in human cells for IF (https://www.sigmaaldrich.com/GB/en/product/mm/05636i) .anti-RAD51 (Millipore Sigma, PC130) was validated in Brendel, V., et al. 1997. J. Mol. Evol. 44, 528 and Boulikas, T., et al. 1997. Anticancer Res. 17, 843.It was also validated by manufacturer in human cells for IF (https://www.merckmillipore.com/GB/en/product/Anti-Rad51-Ab-1-Rabbit-pAb,EMD_BIO-PC130#documentation). anti-RPA32 (Cell Signalling technology, 2208) was validated in Wang et al. Cell Death Dis 9, 923 (2018) and Dharm at al. J Cell Biol 6 November 2017; 216 (11): 3521–3534. It was also validated by manufacturer in human cells for IF (https://www.cellsignal.co.uk/datasheet.jsp?productId=2208&images=1). anti-phospho-RPA32 (S4/S8) (Abcam, ab87277) was validated in Shengqin et al. Nucleic Acids Research, Volume 40, Issue 21, 1 November 2012, Pages 10780–10794. It was also validated by manufacturer in human cells for IHC (https://www.abcam.com/rpa32rpa2-phospho-s4-s8-antibody-ab87277.html). To validate Hel308 (Novus Biologicals NBP1-91842, lot#A91998) specificity, western blot was performed in wild type, HELQ knockout and HELQ siRNA treated cells where single band was detected in only wild type cells.

Eukaryotic cell lines

Policy information about cell lines

Cell line source(s)

We used sf9 insect cells for expression of recombinant proteins available from The Francis Crick Institute, London, UK and these cells are available on request. We used U2OS cells integrated with either DRGFP or SAGFP reporters gifted by Dr. Maria Jasin from Memorial Sloan Kettering Cancer Center New York, NY USA. U2OS cells with integrated SCR reporter were gifted by Dr. Ralph Scully from Harvard Medical School Boston, MA USA. U2OS-DRGFP, U2OS-SA: generated from U2OS cell line ATCC HTB-96. Specifically, Gunn A, Stark JM (2012) I-SceI-based assays to examine distinct repair outcomes of mammalian chromosomal double strand breaks. Methods Mol Biol 920: 379–391. pmid:22941618. U2OS-EJDR: generated from U2OS-DRGFP cell line Bindra RS, Goglia AG, Jasin M, Powell SN. Development of an assay to measure mutagenic non-homologous end-joining repair activity in mammalian cells. Nucleic Acids Res. 2013 Jun;41(11):e115. doi: 10.1093/nar/gkt255. Epub 2013 Apr 12. PMID: 23585275; PMCID: PMC3675474. U2OS-SCR: generated from U2OS cell line ATCC HTB-96 Chandramouly G, Kwok A, Huang B, Willis NA, Xie A, Scully R. BRCA1 and CtIP suppress long-tract gene conversion between sister chromatids. Nat Commun. 2013;4:2404. doi: 10.1038/ncomms3404. PMID: 23994874; PMCID: PMC3838905

Authentication

Cell lines were not authenticated.

Mycoplasma contamination

All human cell lines tested negative for mycoplasma contamination. sf9 cells were not tested for the contamination.

Commonly misidentified lines
(See ICLAC register)

No commonly misidentified lines were used.
